# Cryo-EM structure of the highly atypical cytoplasmic ribosome of *Euglena gracilis*

**DOI:** 10.1093/nar/gkaa893

**Published:** 2020-10-22

**Authors:** Donna Matzov, Masato Taoka, Yuko Nobe, Yoshio Yamauchi, Yehuda Halfon, Nofar Asis, Ella Zimermann, Haim Rozenberg, Anat Bashan, Shashi Bhushan, Toshiaki Isobe, Michael W Gray, Ada Yonath, Moran Shalev-Benami

**Affiliations:** Department of Structural Biology, Weizmann Institute of Science, Rehovot 7610001, Israel; Department of Chemistry, Graduate School of Science, Tokyo Metropolitan University, Minami-osawa 1-1, Hachioji-shi, Tokyo 192-0397, Japan; Department of Chemistry, Graduate School of Science, Tokyo Metropolitan University, Minami-osawa 1-1, Hachioji-shi, Tokyo 192-0397, Japan; Department of Chemistry, Graduate School of Science, Tokyo Metropolitan University, Minami-osawa 1-1, Hachioji-shi, Tokyo 192-0397, Japan; Department of Structural Biology, Weizmann Institute of Science, Rehovot 7610001, Israel; Department of Structural Biology, Weizmann Institute of Science, Rehovot 7610001, Israel; Department of Structural Biology, Weizmann Institute of Science, Rehovot 7610001, Israel; Department of Structural Biology, Weizmann Institute of Science, Rehovot 7610001, Israel; Department of Structural Biology, Weizmann Institute of Science, Rehovot 7610001, Israel; School of Biological Sciences, Nanyang Technological University, Singapore; Department of Chemistry, Graduate School of Science, Tokyo Metropolitan University, Minami-osawa 1-1, Hachioji-shi, Tokyo 192-0397, Japan; Department of Biochemistry and Molecular Biology and Centre for Comparative Genomics and Evolutionary Bioinformatics, Dalhousie University, Halifax, Nova Scotia, Canada B3H 1X5; Department of Structural Biology, Weizmann Institute of Science, Rehovot 7610001, Israel; Department of Structural Biology, Weizmann Institute of Science, Rehovot 7610001, Israel

## Abstract

Ribosomal RNA is the central component of the ribosome, mediating its functional and architectural properties. Here, we report the cryo-EM structure of a highly divergent cytoplasmic ribosome from the single-celled eukaryotic alga *Euglena gracilis*. The *Euglena* large ribosomal subunit is distinct in that it contains 14 discrete rRNA fragments that are assembled non-covalently into the canonical ribosome structure. The rRNA is substantially enriched in post-transcriptional modifications that are spread far beyond the catalytic RNA core, contributing to the stabilization of this highly fragmented ribosome species. A unique cluster of five adenosine base methylations is found in an expansion segment adjacent to the protein exit tunnel, such that it is positioned for interaction with the nascent peptide. As well as featuring distinctive rRNA expansion segments, the *Euglena* ribosome contains four novel ribosomal proteins, localized to the ribosome surface, three of which do not have orthologs in other eukaryotes.

## INTRODUCTION

Ribosomes are macromolecular ribonucleoprotein (RNP) assemblies that function together with transfer RNA (tRNA) in translating messenger RNA (mRNA) into protein. Throughout the three domains of life, ribosomes are highly conserved in composition, structure and sequence (1). Phylogenetic analysis of both ribosomal small subunit (SSU) and large subunit (LSU) RNAs (rRNAs) (2) and proteins (RPs) (3) has traditionally been used to define evolutionary relationships among organisms. Ribosome structures have been studied at the molecular level for the past three decades, primarily through X-ray crystallography, with high-resolution three-dimensional (3D) structures published for SSU, LSU and assembled ribosomes from various prokaryotes and eukaryotes (1,4). In recent years, the revolution in electron cryo-microscopy (cryo-EM) has opened a new era in ribosome research, greatly expanding the phylogenetic coverage and extending it to the ribosomes of eukaryotic organelles, mitochondria (5–7) and chloroplasts (8). These studies have revealed an evolutionarily conserved architecture, with a universal functional core where the peptidyl transferase center (PTC) and the decoding center (DC) are formed by rRNA (9).

**Figure 1.**
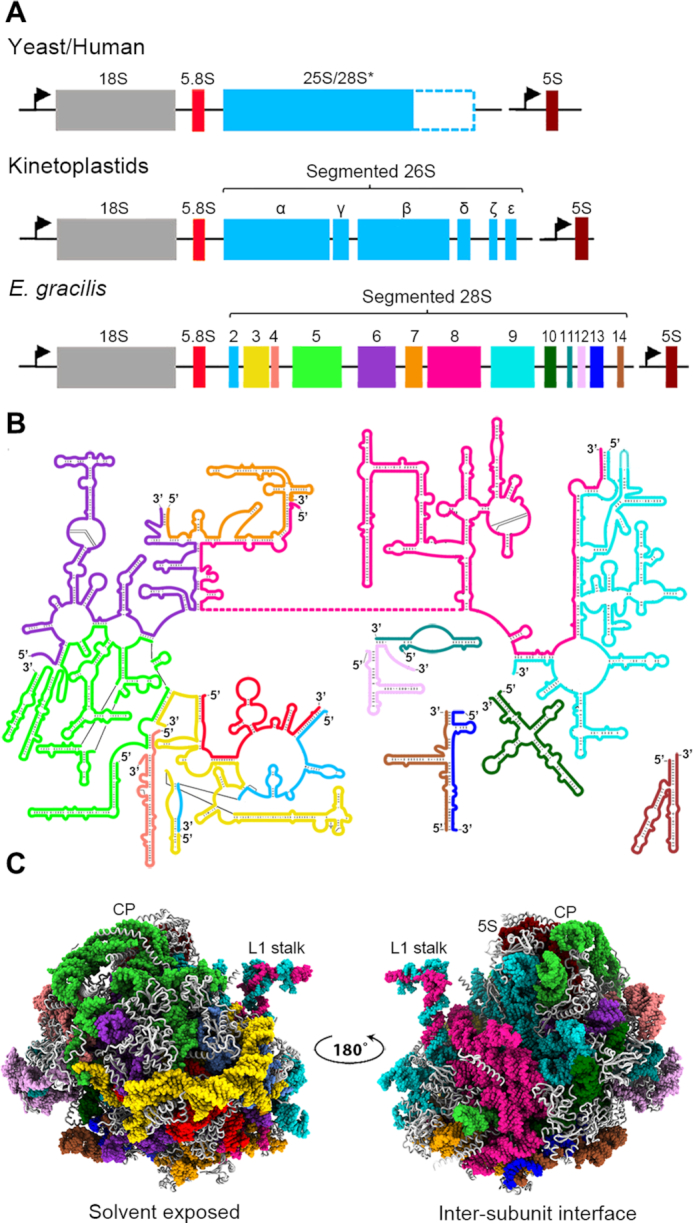
The LSU RNA in *Euglena* is oddly fragmented. In most eukaryotes, the cytoribosome contains three RNA species (18S in the SSU, 5.8S and 25S-28S in the LSU) that are co-transcribed from a joint promoter upstream of the 18S rRNA gene (arrow) and processed into their mature forms by removal of internal transcribed spacers (ITSs). A fourth species (5S, in the LSU) is separately transcribed. In kinetoplastids, the 26S rRNA is further cleaved during maturation of the primary transcript to give six separate fragments (α, γ, β, δ, ζ and ϵ), whereas in *Euglena* the 26S rRNA is fragmented into 13 separate pieces (species 2–14). (**A**) rRNA-encoding operons in yeast, human, kinetoplastids and euglenids. (**B**) Secondary structure map of the *Euglena* LSU rRNA. (**C**) Three-dimensional (3D) model of the *Euglena* LSU. Colors in B and C are as indicated in A. Central protuberance (CP), 5S rRNA and L1 stalk are labeled.

Eukaryotic cytoplasmic ribosomes (cytoribosomes) typically contain four rRNA species: an 18S-sized component in the SSU and 25S-28S, 5.8S and 5S rRNAs in the LSU (Figure 1A). The 18S rRNA and complex of 5.8S:25S-28S rRNAs (the latter referred to as ‘LSU rRNA’) have characteristic secondary structures in which highly conserved regions are interspersed with regions that vary widely in length and sequence (variable regions) and that are mainly responsible for differences in the size of orthologous rRNAs. In some eukaryotes (e.g. human), variable regions have expanded greatly in length in the course of evolution and in these instances are known as expansion segments (ESs) (1,10). These long ESs extend beyond the RP layer that encapsulates the constituent rRNAs and in some cases have been shown to mediate regulatory mechanisms that control translation (11).

Non-conventional cytoribosomes have been described in which the LSU rRNA undergoes additional processing leading to the production of a number of smaller fragments in addition to the 5.8S rRNA. In these, the functional LSU rRNA is naturally fragmented, with the rRNA pieces held together by non-covalent interactions, principally complementary base pairing (12,13). For example, in kinetoplastid protozoa (genera such as *Trypanosoma*, *Leishmania* and *Crithidia*), the 26S rRNA is further cleaved into six fragments (14–18) (Figure 1A). This processing occurs via the removal of internal transcribed spacers (ITSs) in addition to the one separating the 5.8S and 26S coding regions (15).

An extreme case of LSU rRNA fragmentation is found in the eukaryotic alga *Euglena gracilis*, a member of Euglenozoa, the clade that also contains parasitic kinetoplastid genera. In *Euglena*, single rDNA units are encoded in an 11.3-kb covalently closed circular, extrachromosomal plasmid-like DNA (19,20). Processing of pre-rRNA liberates the traditional mature SSU and 5.8S rRNAs, but further cleavage events split the 26S rRNA species into 13 separate pieces via removal of additional ITSs (21) (Figure 1A). Functional domains of LSU rRNA are distributed among these rRNA segments, which have the potential to base pair with one another to reconstitute the overall LSU rRNA secondary structure (22,23) (Figure 1B). Like rRNAs in other eukaryotes, *Euglena* rRNAs are post-transcriptionally modified, with complete maps of modified nucleosides demonstrating that the LSU rRNA (i.e. 5.8S rRNA plus the 13 pieces comprising the 28S rRNA) is substantially more modified than other characterized LSU rRNAs (24). To ascertain how these unusual structural features of highly fragmented LSU rRNA and extremely high level of modification are accommodated within the *Euglena* cytoribosome, and what their functional implications might be, we have determined its 3D structure by cryo-EM (Figure 1C, Supplementary Figures S1 and S2).

## MATERIALS AND METHODS

### Purification of *Euglena* cytoribosomes

*E. gracilis* was cultured in modified Hutner's Low pH *Euglena* Medium [(NH_4_)_2_HPO_4_ 1 g/l, KH_2_PO_4_ 1 g/l, MgSO_4_ 0.2 g/l, sodium citrate 0.8 g/l, CaCl_2_ 0.02 g/l, Fe_2_(SO_4_)_3_.*n*H_2_O 3 mg/l, MnCl_2_.4H_2_O 1.8 mg/l, Co(NO_3_)_2_.6H_2_O 1.3 mg/l, ZnSO_4_.7H_2_O 0.4 mg/l, sodium molybdate 0.2 mg/l, CuSO_4_.5H_2_O 0.2 mg/l, thiamine hydrochloride 0.01 mg/l, vitamin B12 0.0005 mg/l, phosphoric acid to pH 3.5 and absolute ethanol 2.8 ml/l] at 25°C. Cells were harvested at OD_600_ = 1 [4°C, 6000 × *g*, 20 min], washed 3× in resuspension buffer [20 mM HEPES–KOH pH 7.6, 40 mM KOAc, 10 mM Mg(OAc)_2_ and 250 mM sucrose] and resuspended in buffer A [20 mM HEPES–KOH pH 7.6, 40 mM K(OAc), 10 mM Mg(OAc)_2_, 250 mM sucrose, 5 mM β-mercaptoethanol, EDTA-free protease inhibitor cocktail tablet (1 per 50 ml, Roche) and a 1:40 dilution of RNasin U (Promega)]. Cell disruption was performed by French press [5000 psi, 4°C] followed by centrifugation steps designed to remove cell debris, chloroplasts and mitochondria; these include 15 min at 1100 × *g* followed by 30 min at 26 000 × *g*, both performed at 4°C. The clarified supernatant was gently loaded onto a 1.1 M sucrose cushion in Buffer B [20 mM HEPES–KOH pH 7.6, 150 mM KOAc, 10 mM Mg(OAc)_2_, 1.1 M sucrose and 5 mM β-mercaptoethanol] and centrifuged at 116 00 × *g* at 4°C for 16.5 h. The ribosome-enriched pellet was resuspended at 4°C in Buffer C [20 mM HEPES–KOH pH 7.6, 150 mM KOAc, 10 mM Mg(OAc)_2_ and 5 mM β-mercaptoethanol] and centrifuged at 10 000 × *g* for 10 min to eliminate residual chloroplast contamination. The clear supernatant was then subjected to a 15–30% sucrose gradient centrifugation in Buffer C [112 700 × g, 11 h, at 4°C in an SW28 rotor, Beckman]. The 80S peak was collected and centrifuged at 320 000 × *g* for 12 h at 4°C. The pellet was further resuspended in Buffer D [20 mM HEPES–KOH pH 7.6, 100 mM KOAc, 10 mM Mg(OAc)_2_, 10 mM NH_4_OAc and 1 mM DTT] and centrifuged for 1.5 h at 245 000 × *g*, to remove excess sucrose. Finally, the ribosome pellet was resuspended in Buffer D and stored at –80°C at a final concentration of 10 mg/ml until further use.

Ribosome complexes with mRNA and tRNA molecules were assembled by sequential addition of a programmed mRNA fragment (**CACC**AUGUUCAAA, GE Dharmacon) containing a kinetoplastid-specific Kozak sequence (highlighted in bold (25,26)), a P-site start codon (AUG, underlined) and an A-site Phe codon (UUC), P-site tRNA^fmet^ (*Escherichia coli*, Sigma), A-site tRNA^phe^ (*E. coli*, Sigma) and a synthetic derivative of paromomycin (compound 3 in (27)) at 1:100:5:5:100 stoichiometric ratio. Complex assembly was performed at 25°C in ribosome conservation buffer [20 mM HEPES–KOH pH 7.6, 100 mM KOAc; 10 mM Mg(OAc)_2_, 10 mM NH_4_OAc, 2 mM β-mercaptoethanol and 1:40 dilution of RNAsin U (Promega)] with relaxation times of 30 min after the addition of each complex component. Ribosome final concentration was 250 nM.

### Cryo-EM data collection and refinement of *E. gracilis* cytoribosome structure

Ribosome samples (3.5 μl) were applied to glow-discharged holey carbon grids (Quantifoil R2/2) coated with a continuous thin carbon film. The grids were blotted and plunge-frozen using a Vitrobot Mark IV (Thermo-Fischer Scientific). Cryo-EM micrographs were collected at liquid nitrogen temperature on a Titan Krios electron microscope (Thermo-Fischer Scientific) operating at 300 kV. Micrographs were recorded on a Falcon 2 direct electron detector (Thermo-Fischer Scientific) at a nominal magnification of 133K, with a pixel size of 1.05 Å/pixel and a dose rate of ∼1.52 electrons/Å^2^/s. Defocus values ranged from 1.5 to –3.5 μm. Movies were patched-framed-motion-corrected and dose-weighted using Motioncor2 (28). CTFFIND-3 (29) was used for estimation of the contrast transfer function parameters, and RELION-3.0 (30) for downstream image processing steps. Semi-automatic particle picking followed by reference-free 2D classification resulted in a 325 727 particle count that was then subjected to unsupervised 3D classification using a 60-Å lowpass filtered cryo-EM map of the *L. donovani* ribosome (EMD-7024 and EMD-7025) (31) as initial reference. All the classes appeared to contain well-formed 80S particles, therefore the entire particle pool was used for auto-refinement with RELION 3.0. To further improve map quality, especially in the tRNA region, the signal of the already aligned particles, excluding the tRNA binding pocket, was subtracted. Then the remaining particles were subjected to a second 3D classification, which allowed us to identify ribosomes that contained all three tRNAs. Only these particles were subjected to CTF refinement, particle polishing and 3D-refinement to reconstruct a 3.08-Å map. The resulting 3D map was then subjected to a cycle of multi-body refinement (32) using separate masks for the LSU, SSU-head and SSU-body, producing maps at 3.0, 3.02 and 3.13 Å resolutions, respectively. Averaged map resolutions were determined using the gold-standard FSC = 0.143 criterion as implemented in Relion3 and M-triage as implemented in Phenix (33) (Supplementary Figure S1). Local resolutions were estimated using Resmap (34) (Supplementary Figure S2).

### Model building and refinement of the *E. gracilis* cytoribosome structure

Model building of rRNA and RPs was performed combining template-guided and *de novo* model building in COOT (35). The coordinates of the *L. donovani* ribosome (PDB codes 6AZ1 and 6AZ3 (31)) were used as an initial template for model building and were docked onto EM-maps using UCSF chimera (36). *De novo* tracing of rRNA and proteins was also performed in COOT. RNA modifications were manually modeled with coordinates and library files for the modified residues were generated through PHENIX.Elbow (37). Model refinement was performed using an iterative approach including real space refinement and geometry regularization in COOT, followed by real space refinement using the PHENIX suite (33). The final model was validated using MolProbity (38).

### Inference and annotation of ribosomal proteins

The coding and amino acid sequences of cytRPs and associated proteins were inferred from unpublished in-house *Euglena gracilis* transcriptomes and verified by publicly available sequence data available through the Marine Microbial Eukaryotic Transcriptome Sequencing Project, MMETSP (39). *E. gracilis* transcripts were clustered using Trinity (40). We employed BLAST with human cytRPs as heterologous queries to identify transcripts containing the orthologous *Euglena* proteins. In this organism, multicistronic protein-coding transcripts are processed by cleavage and addition of a 5′ conserved spliced leader (SL) sequence to the cleavage products (41). Many of the cytRP transcripts we retrieved contained at least a partial SL, which served to precisely identify the N-terminus of the encoded protein. In cases where a SL sequence was not evident, the N-terminus was assigned based on alignment with orthologous cytRPs from other eukaryotes.

### Analysis of the *E. gracilis* ribosomal proteins by nanoflow LC-MS and –MS/MS

RPs were extracted from purified *E. gracilis* 80S ribosomes and separated by reversed-phase LC on a PLRP-S 1000Å column (2.0 × 100 mm, 10 μm particles, Agilent Technologies). Approx.10 μg RP sample was applied on the column and eluted with a 30-min linear gradient of 10–40% (v/v) acetonitrile in 0.1% trifluoroacetic acid at a flow rate of 100 μl/min. The RPs sample before LC separation (1 μg) or the eluate containing the RP component (50 μl) was adjusted at pH 8, digested with 0.25 μg trypsin overnight at 37°C, and the resulting digest was analyzed by a direct nanoflow LC–MS/MS system equipped with a hybrid quadrupole-orbitrap mass spectrometer (Q Exactive, Thermo Scientific) as described (42). In brief, the tryptic digest was separated on a reversed-phase tip column (100 mm i.d. × 120 mm, Mightysil-C18, 3 mm particles, Kanto Chemical) by a 17 min linear gradient of 0–35% acetonitrile in 0.1% (v/v) formic acid at a flow rate of 100 nl/min. Full MS scans were acquired with a resolution of 35 000. The 10 most intense ions were fragmented under the data-dependent mode by collision-induced dissociation with normalized collision energy of 25. The MS/MS scans were acquired with a resolution of 17 500. The MS data were converted to the MASCOT generic format with the Proteome Discoverer software (Thermo Scientific, version 1.1). The files were processed with the MASCOT algorithm (version 2.3.2., Matrix Science Ltd.) to assign peptides using an in-house database that contains translation in all six reading frames without regard to the length of open reading frame or translation initiation/termination codons. For the search parameters, we set the variable modifications for acetylation (protein N-terminus) and oxidation (Met). The maximum missed cleavage was set at 3 with a peptide mass tolerance of ±15 ppm. Peptide charges from +2 to +4 states and MS/MS tolerances of ±0.8 Da were allowed. Peptides were identified based on the MASCOT definitions. All results of peptide searches were extracted from the Mascot DAT files using the STEM software (43,44). Protein MS data are summarized in Supplementary Table S4.

### Preparation of the *E. gracilis* rRNA and RNase digestion for MS analysis

*E. gracilis* rRNAs were extracted from the purified 80S ribosome samples. An aliquot of 100 μg rRNA was mixed with 800 μl of ISOGEN reagent (Nippon Gene) and passed through a 23-gauge needle 100 times. The sample was further mixed with 200 μl of chloroform, centrifuged at 10 000 × *g* for 15 min at 4°C, and the resulting upper phase (∼500 μl) was mixed with a glycogen solution (0.5 μl, 20 mg/ml) and isopropanol (500 μl) to precipitate the rRNAs. The rRNAs were collected by centrifugation, dissolved in RNase-free water, and stored at −80°C until use.

Purified rRNA fragments were further separated by the reversed-phase LC on a PLRP-S 4000 Å column (4.6 mm i.d. × 150 mm, 10 μm particles, Agilent Technologies) as previously described (45). In brief, ∼10 μg total rRNA were loaded on the column followed by elution at 60°C with a 120-min linear gradient of 11–13.2% (v/v) acetonitrile in 100 mM trimethylamine acetate buffer (pH 7.0) containing 0.1 mM diammonium phosphate with a flow rate of 200 μl/min. RNA detection was through monitoring the eluate at *A*_260_. For further analysis, the purified rRNA fragments were digested with RNase T1 or RNase A in 100 mM triethylammonium acetate buffer (pH 7.0) at 37°C for 60 min.

### Analysis of rRNA modifications by nanoflow LC-MS/MS

The RNase T1/A digest of LSU6 and LSU8 rRNA (100 femtomole each, *∼*50 ng) were analyzed by a direct nanoflow LC–MS system as previously described (45). In brief, the RNase-digested samples were applied on a reversed-phase Develosil C30-UG tip column (150 μm i.d. × 120 mm, 3 μm particles; Nomura Chemical Co., Ltd.) equilibrated with solvent A (10 mM trimethylamine acetate buffer, pH 7, containing 10% methanol) and eluted with a 60-min linear gradient from solvent A to 24.5% solvent B (10 mM trimethylamine acetate buffer, pH 7, containing 40% acetonitrile) at a flow rate of 100 nl/min. The eluate was sprayed through a spray-assisting device into a Q Exactive Plus mass spectrometer (Thermo Fisher Scientific) operating at negative ion mode and data-dependent mode to automatically switch between MS and tandem MS under the conditions described (46). The data analysis software Ariadne (46,47) (http://ariadne.riken.jp/) was used for the assignment of the tandem mass spectral data. with the *E. gracilis* rRNA sequence (Gene ID: M12677, X01484, X02031 and X53361) as reference. The Ariadne search parameters were: the maximum number of missed cleavages was one; two methylations per RNA fragment at any residue position were allowed; an RNA mass tolerance of ±5 ppm and a tandem spectral tolerance of ±20 ppm were allowed.

### Determination of the monomethylated RNA nucleoside positional isomers by nanoflow LC–MS/MS/MS

The digest of LSU6 or LSU8 rRNA (400 fmol, c.a. 200 ng) was analyzed by the nanoflow LC–MS system described above. The LC–MS^3^ condition and the processing of resulting spectral data were as described (46). A summary of MS-identified rRNA modifications is presented in Supplementary Table S7.

## RESULTS AND DISCUSSION

### The overall structure of the *Euglena* ribosome

To unravel the architecture of the *E. gracilis* ribosome we purified intact 80S ribosomes and assembled *in vitro* a ternary complex with mRNA and tRNAs. We then acquired cryo-EM data that were primarily processed through several rounds of 2D and 3D classifications. Our initial set of data included 325,727 particles, yielding a 3.23-Å reconstruction of the entire ribosome (Supplementary Figure S1, Table S1). Inspection of the resulting map indicated three tRNA molecules and an mRNA bound to the 80S particle; however, while E-site tRNA was clearly visualized, the densities for P-site and A-site tRNAs were barely apparent, implying either high flexibility or partial occupancy of the sites across the particle population (Supplementary Figure S1). To resolve the different populations, we focused our classification on tRNA and mRNA by applying a soft mask around this region, followed by signal subtraction of other ribosomal parts. We then subjected the particles to 3D classification, and obtained two major particle subsets: one with E-site tRNA only (38% of total particles), and the other (54% of total particle count) with all three tRNAs bound (Supplementary Figure S1). We next selected particles within the latter class, which demonstrated the most stable tRNA densities (42% of total particle count, 141 860 particles) and reconstructed a map for the whole ribosome particle. In this map, the densities for A- and P-site tRNAs were significantly improved, as was the overall map quality, although no significant change was observed in the averaged map resolution (Supplementary Figure S1C-D). Multibody refinement followed by particle polishing resulted in an improved density map with an overall resolution of 3 Å for the entire particle, and 3.0, 3.02 and 3.13 Å for the LSU, SSU-body and SSU-head, respectively (Supplementary Figure S1–S2; Table S1).

**Figure 2.**
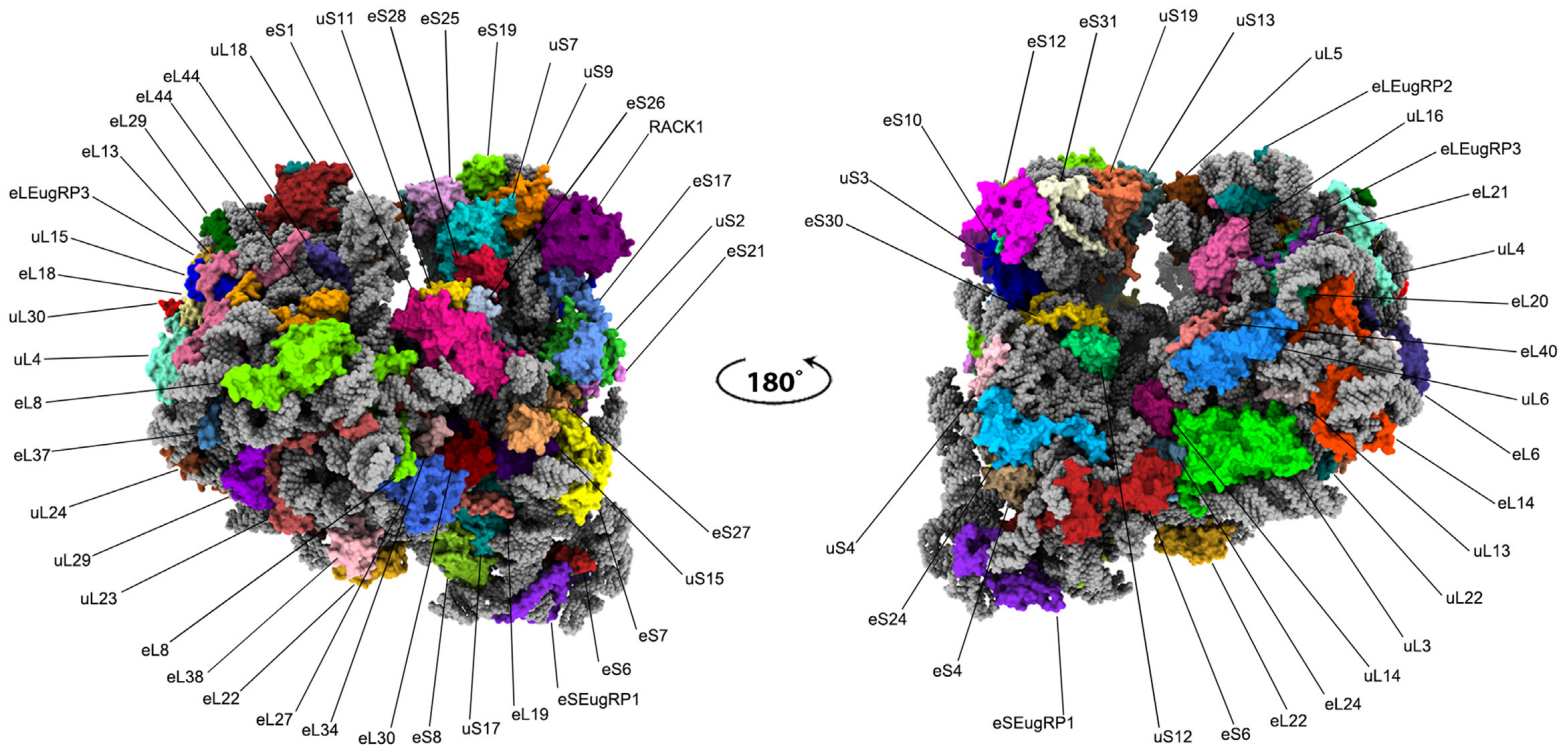
RP profile in the *Euglena* cytoribosome. Overall model of the *Euglena* ribosome with rRNA presented as grey spheres and RPs in color surface.

**Figure 3.**
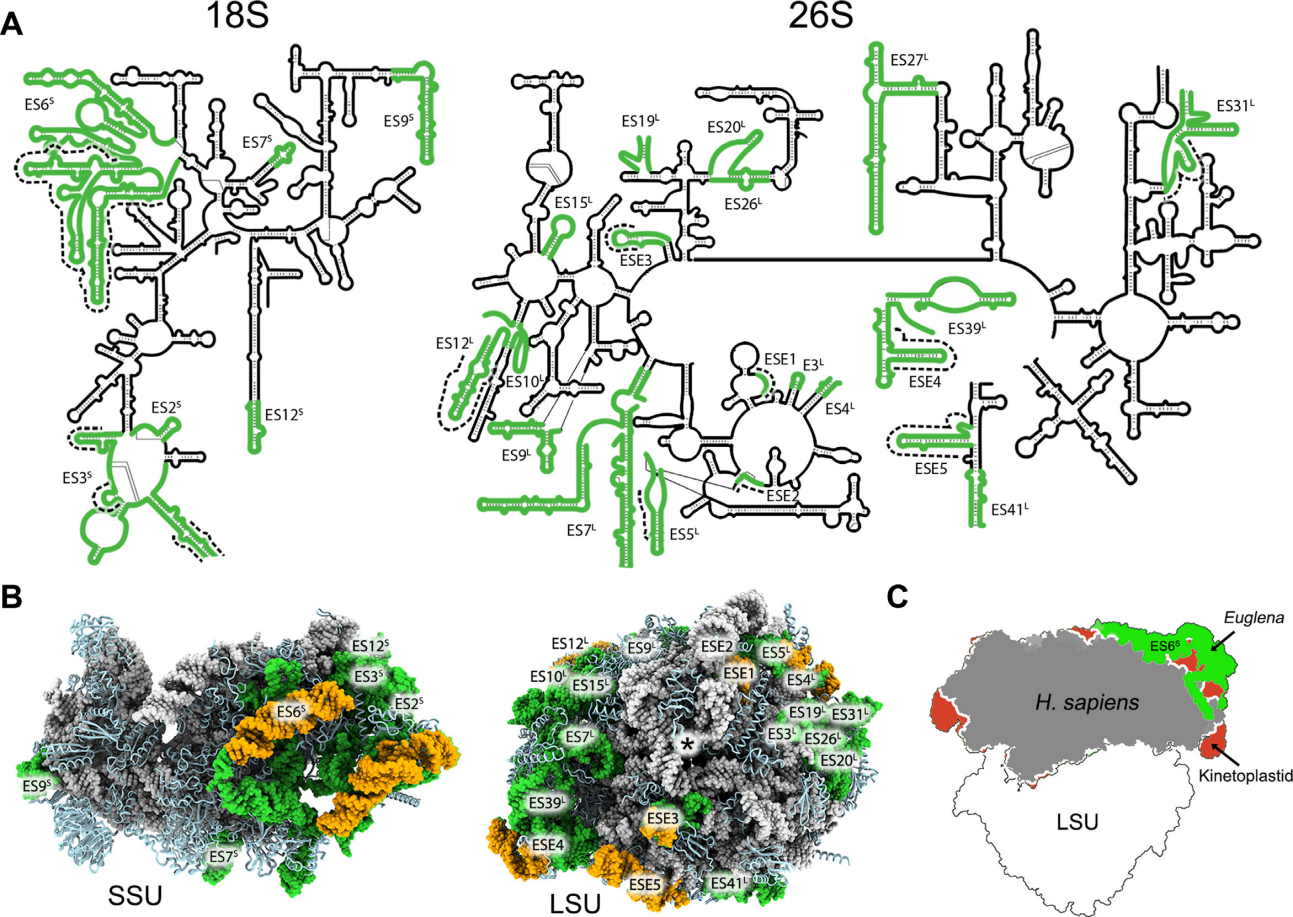
RNA expansion segments (ESs) in the *Euglena* ribosome. (**A**). 2D maps showing the location of ESs (green) in the rRNA secondary structures. These ESs are spread out in both the SSU (left) and the LSU (right) rRNAs. Segments unique to *Euglena* are indicated by a dashed black line. (**B**) 3D representation of ESs is at the bottom of the panel, with rRNA colored grey; RPs depicted in light blue and ESs in green. *Euglena*-specific extensions are in yellow. In the LSU, exit tunnel is marked with an asterisk. (**C**) The foot region in the *Euglena* SSU is significantly enlarged compared to other eukaryotes (*Euglena–*green, human–gray, kinetoplastids–red), due to the exceptionally large ES6S in this organism. Structures used for comparison are PDB ID 4UG0 for human and PDB ID 5OPT for kinetoplastids (*T. cruzi*).

The final map allowed us to build, refine and validate the complete model of the *Euglena* ribosome, including all sixteen rRNA components (18S, 5.8S, 5S and the thirteen segments comprising the 28S rRNA), three tRNAs, an mRNA and a total of 34 RPs for the SSU and 41 for the LSU (Figures 1 and 2, Supplementary Figure S3 and Table S2). Notable structural changes are most prominent in the SSU, which exceeds the size of a typical 40S particle and bears extended RNA segments in the body region, as well as harboring an additional RP (termed here eSEug1, Figures 2 and 3). For the LSU, all of the rRNA segments were readily visible, indicating that although the rRNA in this ribosome is highly fragmented (22,23), the RNA segments interact extensively to form the universally conserved ribosome backbone (Figure 1, Supplementary Figure S4). Notable differences were observed in the protein composition of the LSU, and include the absence of RP eL28 and the presence of three *Euglena*-specific proteins, denoted here as eLEgr1, eLEgr2 and eLEgr3 (Figure 2).

### *Euglena* LSU rRNA is uniquely fragmented and contains species-specific expansion segments

The LSU rRNA of *Euglena* differs from the typical rRNA in eukaryotes as it is uniquely fragmented into 14 segments (Figure 1). This segmentation occurs post-transcriptionally (48) through the precise excision of internal transcribed spacers (ITSs) by an as-yet unknown mechanism (21). Similar segmentation, albeit to a lesser extent, is also observed in kinetoplastids such as *Leishmania*, *Trypanosoma* and *Crithidia*, such that the cytoplasmic LSU is composed of seven discrete rRNA segments (15,49). Recent structural studies in kinetoplastids (18,50,51) revealed the 3D organization of these highly fragmented ribosomes, indicating that despite the pronounced degree of LSU rRNA segmentation, the overall structural organization of the LSU is highly comparable to that of other ribosomes. The studies further revealed that the rRNA chain ends are clustered to three solvent-exposed regions of the LSU, implying a mechanism in which cleavage events occur late in the ribosome assembly pathway (18,50).

**Figure 4.**
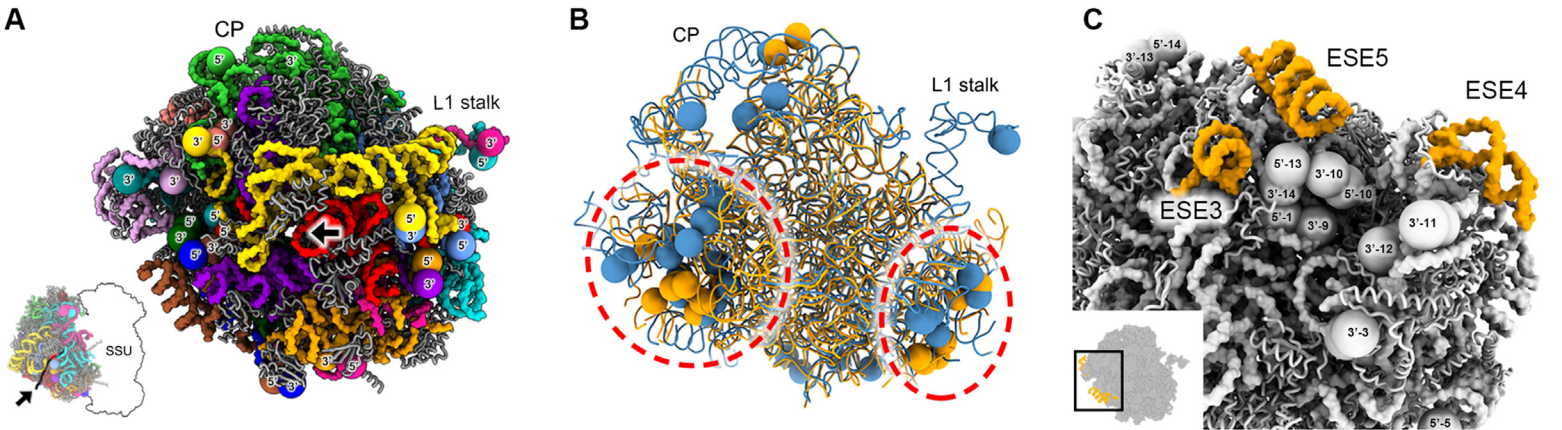
RNA chain ends in the *Euglena* LSU are localized to surface-exposed ribosomal regions. (**A**) A view of the *Euglena* ribosome LSU overviewing the LSU surface. LSU RNA chains are colored in distinct colors, with chain ends represented by spheres. 5′ and 3′ ends are indicated. Nascent protein exit tunnel is indicated with an arrow. L1 stalk and central protuberance (CP) are noted for orientation. (**B**) rRNA chain ends in *Euglena* converge to five focal points on the LSU surface. Two of these foci overlap the position of rRNA segment ends in kinetoplastids. *Euglena* rRNA is colored blue, kinetoplastids yellow. Shared focal points are circled with a dashed red line. Central protuberance (CP) and L1 stalk are labeled. PDB ID 6AZ3 (*Leishmania*) used for alignment with the *Euglena* ribosome. (**C**) Three ESEs localized to the LSU surround a major focal point, at which many rRNA chain ends converge on the ribosome surface. Miniaturized panel in the left bottom corner highlights the location of this region within the complete ribosome.

The cryo-EM structure reveals that, as in kinetoplastids, the *Euglena* LSU shares a similar fold with other eukaryotic ribosomes (Supplementary Figure S4), and although the number of LSU rRNA segments in this ribosome species is doubled compared to kinetoplastids, its RNA chain termini also face the solvent-exposed region, clustering in focal points (Figure 4A) that largely overlap those observed in trypanosomatids (Figure 4B) (50). However, the *Euglena* ribosome harbors additional RNA end clusters that are unique to this organism, two of which are localized in close proximity to the central protruberence (CP) and at the tip of the L1 stalk (Figure 4A). The observation that the rRNA chain ends converge to a few focal centers at the ribosome surface implies that in *Euglena*, rRNA processing likely occurs following assembly with RPs during the late stages of ribosome biogenesis. The overlap with the same focal positions in trypanosomatids implies a common processing mechanism between trypanosomatids and euglenids, in which the enzymes that participate in these events trim the rRNA chain ends after folding and assembly. However, the detailed mechanism by which ITSs are processed and how they function in these organisms remain major open questions in the field.

In addition to the unique rRNA segmentation, and similar to other eukaryotes, the *Euglena* ribosome surface is highly enriched with rRNA expansion segments (ESs), some of which are *Euglena*-specific (ESEs, Figure 3). Of note is the *Euglena*-specific expansion of ES6^S^ in the SSU, which adds significant volume to the SSU foot region compared to that of other ribosomes (Figure 3C), and which also harbors a Euglenozoa-specific RP, as described below. In the LSU, the *Euglena-*specific rRNA expansion domains (ESE1–5) are clustered in two surface-exposed regions, localized in close proximity to the major rRNA chain end bundles (Figure 4C). Similarly, in *Leishmania* and *Trypanosoma* ribosomes, kinetoplastid-specific expansion segments were found in close proximity to rRNA chain end clusters, suggesting a potential role in ribosome biogenesis in these species (17,50).

### The *E. gracilis* cytoribosomal protein profile differs from that of other eukaryotes

We inferred complete sequences for 33 SSU and 47 LSU cytoribosomal proteins (cytRPs): all but eL28 and including two paralogs for eL15 (Supplementary Figure S5 and Table S3). *Euglena* cytRPs originating from purified ribosome samples were separated by liquid chromatography and subjected to tandem mass spectrometry (LC–MS/MS). The resulting tryptic peptide data combined with a whole transcriptome analysis identified a total of 248 proteins, among which were 76 of the 80 inferred *E. gracilis* cytRPs (Supplementary Table S4).

**Figure 5.**
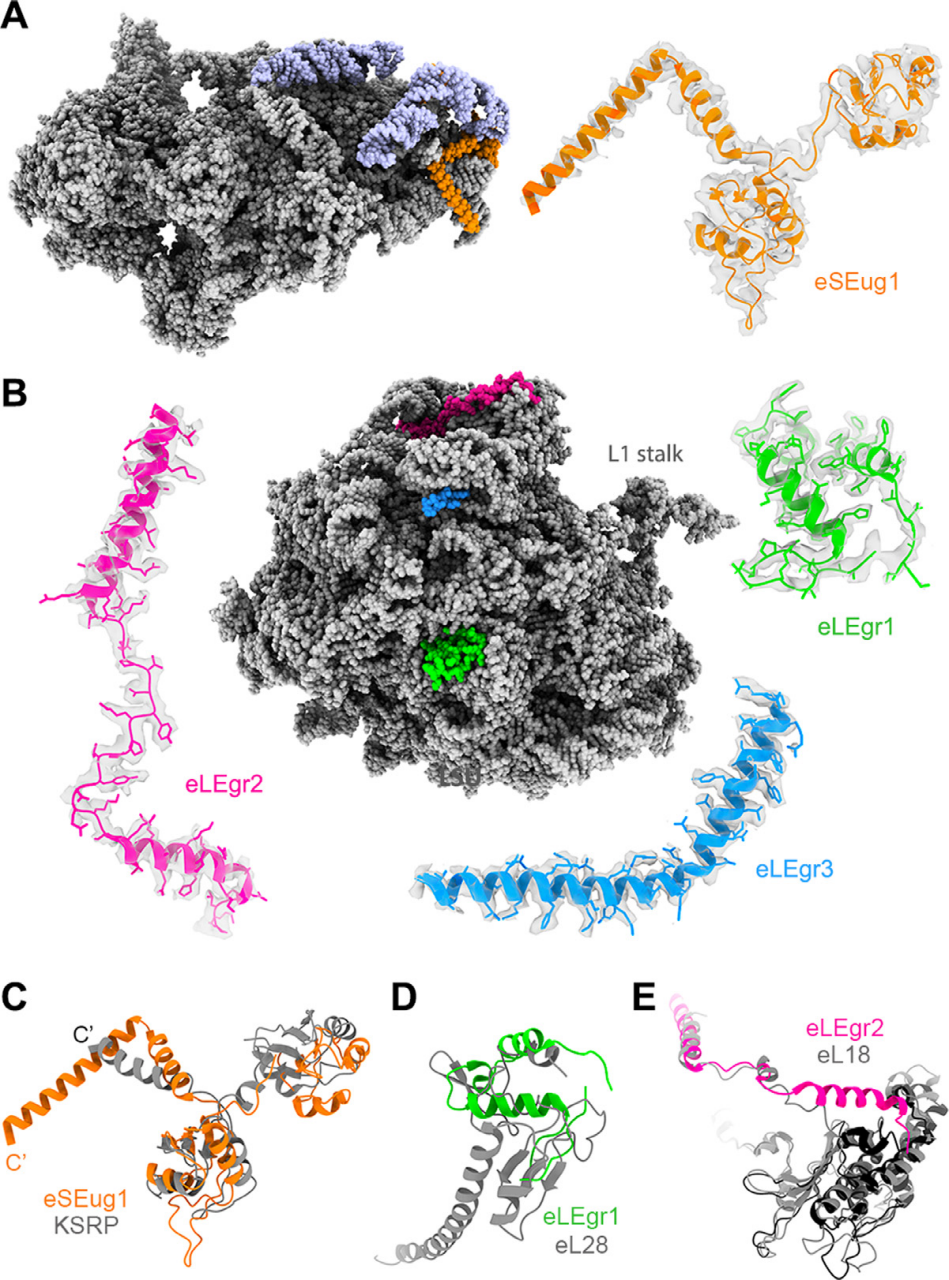
The *Euglena* cytoribosome has four unique proteins. Four unassigned protein densities were identified in the cryo-EM maps of the *Euglena* ribosome. These include a Euglenozoa-specific protein (eSEug1) in the SSU (**A**), and three *Euglena*-specific proteins (eLEgr1–3) in the LSU (**B**). (**C**) eSEug1 (orange) is a homolog of the kinetoplastid-specific RNA-binding protein KSRP described in the ribosomes of trypanosomatids. KSRP of *T. cruzi* is shown in grey (PDB ID 5OPT). eSEug1 is buried within an rRNA expansion segment comprising ES6^S^, which is substantially extended in the Euglenozoa phylum (rRNA expansion segment is highlighted in light purple, (A). (**D**) eLEgr1 (green) localization overlaps the eL28 position in other ribosomes. Notably an eL28 homolog is missing in *Euglena*, and accordingly the overall fold of eLEgr1 has diverged from that of eL28. (**E**) eL18 in *Euglena* is significantly shorter compared to other eukaryotes (black); however, the *Euglena*-specific protein eLEgr2 (magenta) overlaps the C-terminus of eL18 in other eukaryotic ribosomes. In panels (C)–(E), comparison is with the *T. cruzi* ribosome (gray), PDB ID 5T5H.

Our cryo-EM map allowed the building of the inferred RP structures of the *Euglena* cytoribosome (Figure 2 and Supplementary Table S2). However, additional unassigned densities were noted suggesting the presence of novel proteins, one within the SSU and three in the LSU. In the SSU the differential density was localized to the foot region that is significantly larger in *Euglena*, due to the expansion of ES6^S^ in this species (Figure 3B, C). The protein density was found to overlap a kinetoplastid-specific RP (KSRP) that was previously described in *Trypanosoma* and *Leishmania* (52) and is considered an integral component of the trypanosome SSU (Figure 5A, C and Supplementary Figure S6). A whole transcriptome sequence analysis retrieved a *Euglena* KSRP homolog, corresponding to eSEug1 (Supplementary Figure S6A), and the presence of the protein was also confirmed by MS analysis (Supplementary Table S4). The *E. gracilis* homolog is extended by 25 amino acids at the C-terminus compared to its kinetoplastid counterparts, thus encompassing an additional helical extension that makes further contacts with ES3 rRNA. The latter is longer in kinetoplastids compared to other eukaryotes. As *Euglena* and kinetoplastids belong to the Euglenozoa phylum, we suggest a phylum-related nomenclature for KSRP, namely, eSEug1 (small subunit Euglenozoa-specific ribosomal protein 1).

In the LSU, the three unassigned densities were found on the ribosome surface, corresponding to small proteins 60–70 amino acids long. Two of the observed densities were localized to the CP region and one overlapped the location of eL28 at the surface-exposed region of the LSU (Figure 5B, D and Supplementary Figure S6C, D). Guided by the side chain densities in the cryo-EM map, and in comparison with the MS data in hand, we were able to determine the protein identities and attribute them to the unassigned densities. A comprehensive sequence and structural analysis did not reveal any related homologs and we thus conclude that they are *bona fide Euglena*-specific RPs, which we have named eLEgr1–3.

eLEgr1, which partially overlaps the location of eL28, is significantly shorter than eL28 and the two proteins share limited structural similarity (Figure 5D and Supplementary Figure S6C). Notably, a sequence corresponding to a conventional eL28 homolog could not be found in the deeply sequenced *E. gracilis* transcriptome and sequence similarity between eLEgr1 and eL28 is extremely low. The apparent absence of eL28 was surprising because we readily detected an ortholog in kinetoplastid species such as *T. cruzi*, as well as in other members of Discoba (*Naegleria gruberi*, *Andalucia godoyi*), the eukaryotic supergroup to which Euglenozoa belongs. eL28 functions in most eukaryotes to stabilize ES7^L^-A (53), whereas in kinetoplastids a C-terminal extension of eL28 also stabilizes a unique kinetoplastid rRNA extension, ES42L (Supplementary Figure S6C-D). As both extensions are missing in the *Euglena* ribosome (Supplementary Figure S6C-D) we postulate that *Euglena* carries a primordial version of this protein that was further elaborated later in evolution to stabilize additional rRNA ESs. Notably, eL28 is missing in yeast ribosomes (54), whose LSU rRNA also lacks the same eukaryote-specific extension (Supplementary Figure S6C).

eLEgr2 is localized to the CP region and overlaps the C-terminus of eL18 in other eukaryotic ribosomes (Figure 5B,E). *Euglena* eL18 is shorter compared to its eukaryotic counterparts and, as euglenozoans are considered to have diverged rather early in evolution, we postulate that eLEgr2 was originally produced as an independent protein and later in evolution combined as part of eL18. Alternatively, eLEgr2 might be the result of an eL18 gene fission resulting in a shorter version of this protein and the newly identified protein. Gene fusion and gene fission are considered to be major contributors to the evolution of multi-domain bacterial proteins (55) and are suggested to have played an important role in RP evolution prior to the emergence of the modern ribosome (56–58).

In contrast to eLEgr1–2, eLEgr3, also localized to the CP region, does not overlap any known RP reported so far (Figure 5B and Supplementary Figure S6E). Interestingly, this protein along with eL29 creates a tight network of interactions with ES12^L^ that is extended in *Euglena* compared to other eukaryotes, as well as with ESE5 (Supplementary Figure S6F). This implies that eLEgr3 evolved to stabilize the *Euglena*-specific rRNA expansion segments. Notably, eLEgr3 shares structural similarity with the prokaryotic protein L10, the counterpart of P0 proteins localized to the L7/L12 stalk. However, the localization of the P0/L10 proteins does not correspond to the eLEgr3 density (Supplementary Figure S6G).

In addition to the *bona fide Euglena* RPs, four conserved cytRPs are substantially longer than their counterparts in other eukaryotes (Supplementary Figure S7A-B). These include the SSU RP eS6 that features an extended C-terminus of 25 residues, making additional contacts with ESE6^S^, which is significantly enlarged in *Euglena* (Supplementary Figure S7C). Additionally, LSU RPs eL22 and eL37 have distinctive N- and C-terminal extensions. In eL22, the extended N-terminus faces the SSU, in close proximity to eS8, which forms the ribosomal bridge eB11 (59) (Supplementary Figure S7D). The density for the protein extension is ambiguous within the EM maps due to residual mobility, thus has only been partially modeled in our structure. However, as the N-terminus is positioned towards the SSU, it is very likely that it participates in the formation of a *Euglena-*specific ribosomal bridge. eL37 encompasses an extended α-helical domain that is localized to the solvent-exposed side of the LSU. This extension maintains close contacts with H17, and overlaps its position in other ribosomes. As a result, H17 is ∼40 Å away from the same reported helix in human and kinetoplastid ribosomes (Supplementary Figure S7E). eL6 contains a notable internal insert, ∼40-amino acids long when compared to its eukaryotic counterparts (Supplementary Figure S7F). This extended loop fills a cavity within the LSU that is occupied by an rRNA expansion segment in kinetoplastids.

### The *E. gracilis* ribosome is highly enriched with rRNA modifications

**Figure 6.**
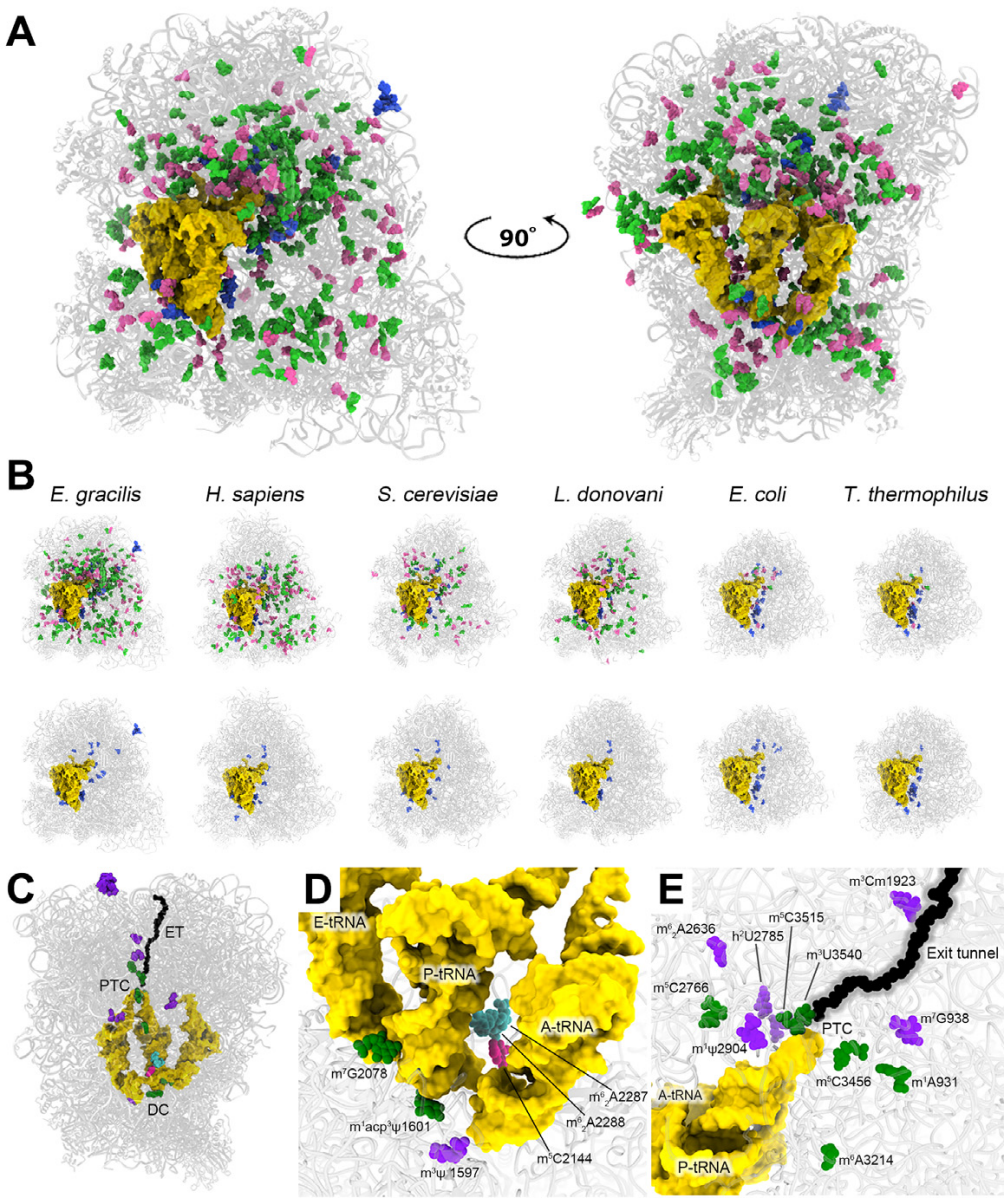
*Euglena* ribosome rRNA modifications. The high-resolution structure obtained in this study allowed the direct visualization of rRNA modifications in the *Euglena* ribosome. An overview of modified residues is shown in (**A**). *O*^2’^-methylated residues (Nm) are highlighted in green, pseudouridine residues in pink, and unique base modifications in blue. (**B**) Modifications of *Euglena* rRNA compared with the homologous human, yeast, kinetoplastid and bacterial rRNAs. Nm residues are in pink, Ψ in green and base modifications in blue. Lower panel in B shows base modifications only. Ribosome extent is represented by the grey background, tRNA and mRNA are depicted in yellow. (**C**) Base modifications in *Euglena* are mostly localized to functional ribosomal regions. (**D**) Six unique base modifications in the 18S rRNA are localized to the SSU–LSU interface and maintain interactions with both mRNA and A- and P-site tRNAs. (**E**) The LSU rRNA of *Euglena* contains 16 unique base modifications, mostly distributed within the PTC and around the protein exit tunnel. Five of these modifications are also conserved in human and yeast ribosomes (green), whereas 12 modifications have only been reported in *Euglena* so far (purple). In (C)–(E), base modifications are colored according to conservation, with universally conserved residues in cyan, modifications of bacterial origin pink, eukaryote-conserved green, and *Euglena*-specific purple.

The *E. gracilis* ribosome is decorated with a total of 349 rRNA modifications, the highest number of ribosomal modifications reported to date (24) (Figure 6, Supplementary Figure S3 and Table S5). Similar to other eukaryotes, most (∼95%) of these modifications correspond to methylations at the *O*^2′^-ribose positions (Nm) and pseudouridines (Ψ). However, compared to other organisms, in *Euglena* these modifications differ both in their unprecedented levels and their overall distribution, which extends far beyond the ribosomal core regions (Figure 6A, B, Supplementary Figure S8 and Tables S5 and S6) (60–63). Of particular interest is the observation that the *Euglena* LSU rRNA is markedly enriched in modifications compared to the SSU rRNA. The greatly enhanced level of post-transcriptional modification of LSU rRNA correlates with the high level of fragmentation of this rRNA species.

Our cryo-EM analysis enabled the direct visualization of 186 Nm residues in the *Euglena* ribosome, revealing their 3D context (Supplementary Table S5). Due to similar geometry, Ψ residues could not be visualized through cryo-EM, but as these residues were identified and mapped in a previous study (24), we were able to use this information to model them into the 3D ribosome structure (Supplementary Figure S8, Table S5). Similar to other organisms, Ψ and Nm are found in both ribosomal subunits and although most of them are present within or in close proximity to the ribosomal functional sites, overall they are rather widely distributed within the constituent rRNAs (Figure 6A, B). A similarly broad distribution, albeit to a lesser extent, was also visualized in ribosomes of *Leishmania* and *Trypanosoma*, which also have segmented LSU rRNAs, and compared with other eukaryotes also have an elevated count of modified residues (31,46,64,65). Considering the role Nm and Ψ residues have been suggested to play in mediating RNA stability (60,66,67), we hypothesize that similar to the trypanosomatids, the relatively large number of rRNA modifications in *Euglena* contribute to the overall stabilization of this oddly fragmented ribosome.

In addition, 22 base-modified residues are present in the *Euglena* ribosome, six in the SSU and sixteen in the LSU (Figure 6C and Supplementary Table S5). Such an elevated level of base modification is also unprecedented in eukaryotic ribosomes characterized so far, with yeast, human and even *Leishmania*, exhibiting at most a total of 12 base-modified residues. Similar to other eukaryotes, the base modifications in the *Euglena* ribosome are mostly assigned to core ribosomal domains and are clustered in conserved key functional sites of the ribosome, such as the decoding center, PTC and exit tunnel, maintaining direct contacts with the ribosomal substrates (Figure 6C–E).

**Figure 7.**
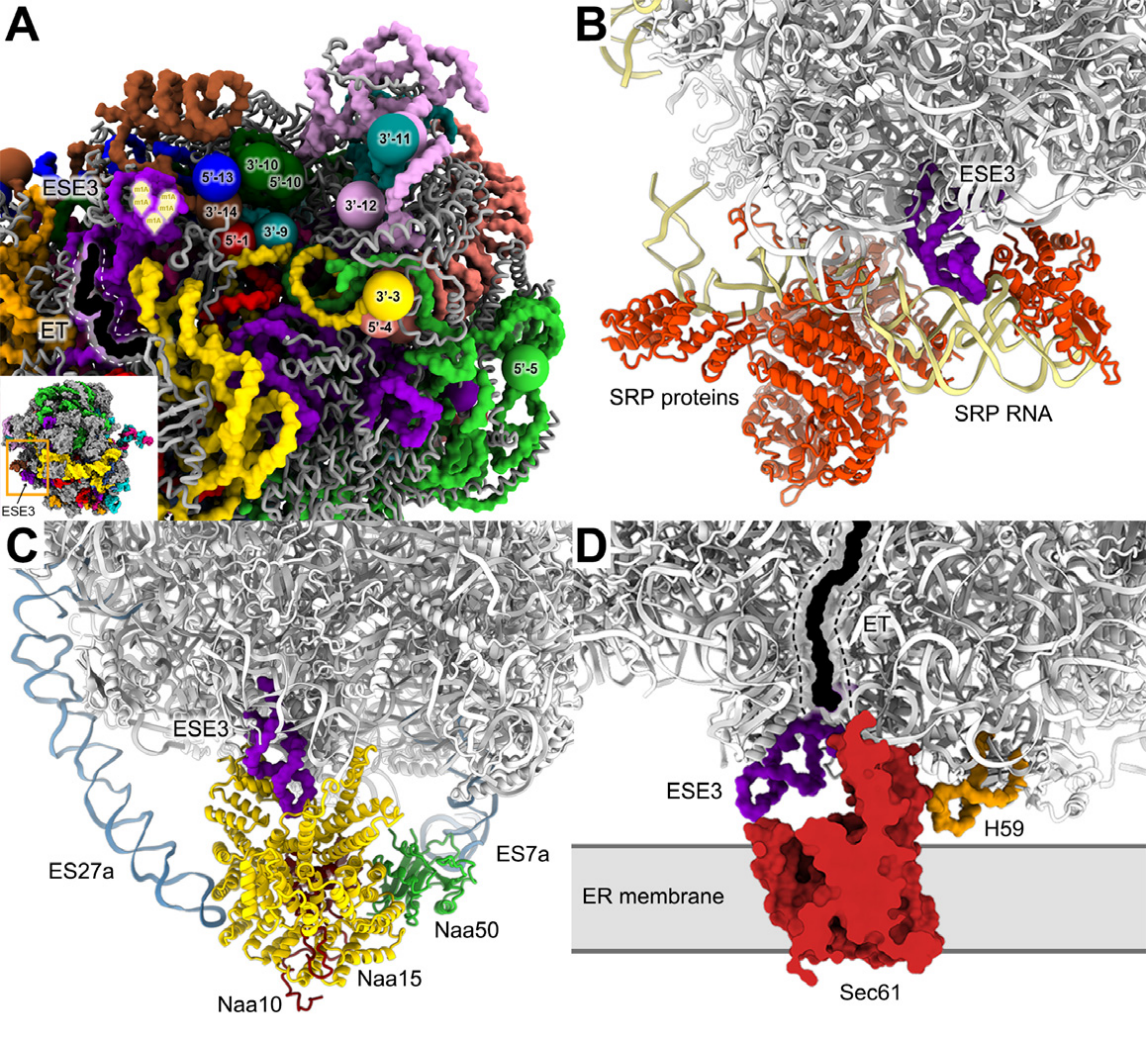
m^1^A residues uniquely localize to a *Euglena*-specific expansion segment. A particularly intriguing feature in the *Euglena* ribosome is the presence of a unique m^1^A-enriched segment localized to a *Euglena*-specific rRNA extension segment (ESE3). (**A**) ESE3 is shown facing a ribosome surface region where a substantial portion of the rRNA segment ends converge. Segment termini are depicted as spheres, with chain numbers and directionality indicated. rRNA chains are colored according to the scheme shown in Figure 1. ESE3 is in purple, with the m^1^A stretch indicated by yellow tags. Protein exit tunnel (ET), localized in close proximity, is in black. (B–D) ESE3 is surface-exposed and is positioned to interact with auxiliary proteins that bind to the nascent peptide and aid in its processing or localization. (**B**) Superposition with the SRP complex indicates that ESE3 fits into a cavity in the SRP RNA, where positively-charged m^1^A residues could potentially bind to the negatively-charged SRP-RNA backbone. The figure was prepared through alignment with the structure of the mammalian SRP complex interacting with the ribosome (PDB ID: 6FRK). (**C**) ESE3 occupies a cavity within Naa15 (yellow), which is a component of the NatA complex that acts to acetylate the N-terminus of nascent peptides. The *Euglena* ribosome is superposed on the NatA complex structure (PDB ID: 6HD7). NatA components Naa10,15 and 50 are colored with red, yellow and green, respectively. Eukaryote-specific ES27a and ES7a, which directly interact with the complex and are highly conserved in eukaryotes, are indicated in blue. (**D**) ESE3 protrudes from the ribosome interface, and due to the methylation of five clustered residues it is positively charged, and thus could potentially interact with the negatively-charged ER membrane. Superposition with the structure of membrane-anchored ribosomes through Sec61 (PDB ID: 3J7R) indicates that ESE3 is in close proximity to the translocon and is indeed positioned to directly contact the membrane. H59 (orange), previously shown to contact the ER membrane, is localized on the other side of Sec61.

Notably, a cluster of five *N*-1-methyladenosine residues—m^1^A 1863, 1864, 1866, 1868 and 1869—exists in the LSU of the *Euglena* ribosome. These modifications were identified through chemical mapping of the *Euglena* LSU rRNA (24) and were further verified through our MS analysis (Supplementary Table S7). Our model indicates that the m^1^A modifications are clustered in a region remote from the ribosome core and localized to a *Euglena-*specific rRNA expansion segment (ESE3, Figure 6C and 7A). This segment was clearly visualized in the unsharpened cryo-EM maps. However, due to helix flexibility; the nucleobase positions could not be assigned; thus, only the backbone of this region has been modeled. Solitary m^1^A residues are known to occur in tRNA and rRNA, in both prokaryotes and eukaryotes (e.g. yeast LSU rRNA (68,69)) and more recently have been reported in mRNA (70–72). However, the cluster of m^1^A in *Euglena* LSU rRNA is so far unprecedented.

m^1^A is known to have a major effect on structure and function by virtue of the fact that it has both a methyl group and a positive charge under physiological conditions (73). As well, m^1^A methyltransferase activity has been found to be crucial for tRNA and ribosome maturation (68,74–76), and mutations in their respective methylation enzymes are associated with human disease (77) and lack of adaptation to changing environmental conditions (78,79). Given the involvement of rRNA modifying enzymes in ribosome biogenesis and RNA folding and stability, it is not surprising that the *Euglena* ribosome, which undergoes an unusual rRNA maturation process, characterized by a high degree of LSU rRNA fragmentation, also exhibits an unusual modification pattern. Notably, the m^1^A stretch in *Euglena* LSU rRNA is potentially able to interact with the most crowded focal point in the *Euglena* ribosome, where 12 LSU rRNA fragment ends converge (Figure 7A). Thus, the five positive charges in the ESE3 m^1^A cluster under physiological conditions might contribute to the neutralization of negatively-charged primary phosphoryl groups at clustered fragment 5′ ends, thus helping to stabilize this region of the ribosome. The close proximity of an m^1^A cluster to a focal point involving almost half of the LSU rRNA fragments might also be an indication of the potential involvement of ESE3 in the recruitment of a methyltransferase implicated in the *Euglena*-specific processing events required for ribosome maturation.

The m^1^A cluster is also in close proximity to the exit path of the nascent peptide, highlighting the possibility that it might function in the mature ribosome for the recruitment of auxiliary proteins or even for maintaining direct contact with the nascent protein itself (Figures 6C and 7A). An exciting possibility would be the recruitment of proteins that participate in chaperone activity of the nascent chain, in its post-translational modulation or in ER translocation for membrane protein translation. Indeed, superposition of the *Euglena* ribosome structure with recently published structures of such auxiliary components revealed that the m^1^A stretch at ESE3 would be positioned to interact with components of the SRP complex through direct stabilization of the SRP-RNA (Figure 7B) (80,81), as well as with modifying enzymes such as NatA (82) that act to acetylate the N-terminus of the nascent peptide chain (Figure 7C). Of note, superposition of the *Euglena* structure with structures of the Sec61-ribosome complex (83) indicate that ESE3 could potentially interact directly with the bilayer membrane (Figure 7D). Assuming the positively charged nature of this modified expansion, it is also plausible that the modified region could directly interact with the negatively-charged membrane.

In summary, the *Euglena* cytoribosome is an unusual RNP, which is distinct from other ribosomes in that it harbours four unique core ribosomal proteins, while its LSU rRNA is highly fragmented. The RNAs in this ribosome species (particularly the LSU rRNA) are also substantially enriched in post-transcriptional modifications, which are spread far beyond the catalytic RNA core, likely contributing to the stabilization of rRNA fragments. A notable feature of this ribosome is a cluster of five m^1^A residues localized to a distinctive LSU rRNA expansion segment on the ribosomal surface and adjacent to the protein exit tunnel. This extension is uniquely positively charged and might serve to stabilize a negatively-charged pocket enriched with rRNA segment ends, and/or to potentially interact with auxiliary proteins that act on the nascent peptide or aid its localization.

## DATA AVAILABILITY

The cryo-EM maps of the *Euglena* cytoribosome have been deposited at the Electron Microscopy Data Bank (EMDB-11232). The atomic model has been deposited in the PDB with accession number 6ZJ3. Annotated *Euglena gracilis* cytRP sequences (CDS and amino acid) have been deposited in GenBank under accession numbers MT583833–MT583918.

## Supplementary Material

gkaa893_Supplemental_File
