# Supplementary material for: Cryo-EM structure of the highly atypical cytoplasmic ribosome of Euglena gracilis
Source: Nucleic Acids Res. 2020 Oct 22;48(20):11750–61. doi: 10.1093/nar/gkaa893 (PMC7672448; doi:10.1093/nar/gkaa893)
Supplement: gkaa893_Supplemental_File [file gkaa893_supplemental_file.pdf]

Supplementary Data

Cryo-EM structure of the highly atypical cytoplasmic ribosome of *Euglena gracilis*

Donna Matzov[1], Masato Taoka[2], Yuko Nobe[2], Yoshio Yamauchi[2], Yehuda Halfon[1], Nofar Asis[1], Ella Zimermann[1], Haim Rozenberg[1], Anat Bashan[1], Shashi Bhushan[3], Toshiaki Isobe[2], Michael W. Gray[4,#], Ada Yonath[1,#] and Moran Shalev-Benami[1,#]

List of contents:

Supplementary Figures S1-S8 Supplementary

Tables S1-S7

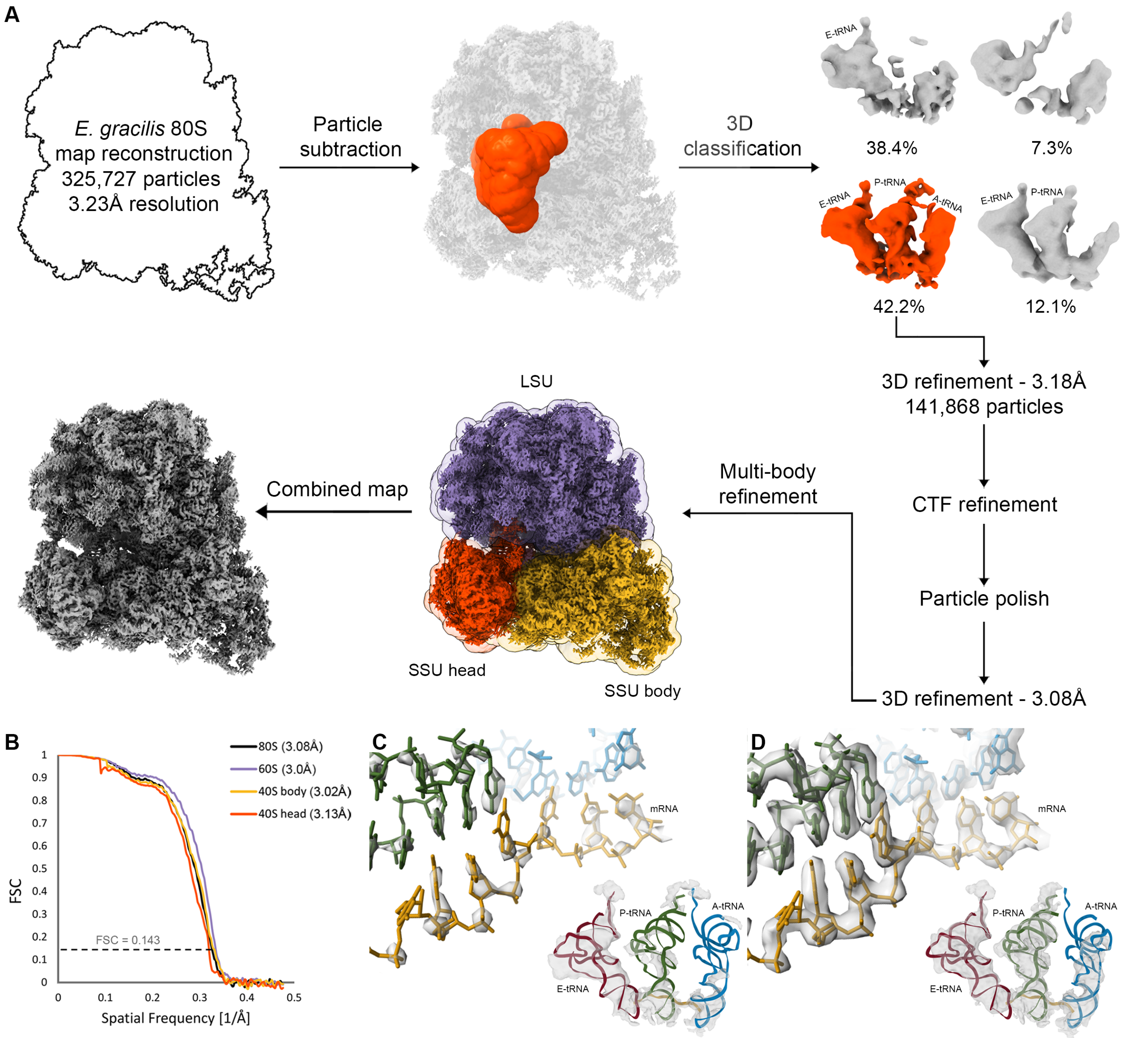

Supplementary Figure S1| Cryo-EM data processing and map reconstruction. (**A**) Schematic representation of EM data processing for the *E. gracilis* ribosomes. Data processing was performed in Relion 3.0 and included motion and CTF correction, particle picking and classification. Initial map reconstruction and post-processing were performed by the 3D refinement algorithm implemented in Relion on the complete 80S particle, indicating high residual mobility of the SSU head domain, along with partial occupancy of tRNAs. Further implementation of signal subtraction and masked classifications followed by multibody refinement with individual masks prepared for the LSU (purple), SSU body (yellow) and SSU head (orange) yielded the reconstruction of the complete 80S particle along with the three tRNA substrates and an mRNA. Final map is presented in the bottom left corner of the panel, FSC curves indicating overall (black) and per-domain (colored) resolutions are presented in (**B**). Comparison of tRNA density in the complete ribosome map before (**C**) and after (**D**) signal subtraction and particle sorting. Unsharpened map contours of tRNA and mRNA are shown in the bottom right corner of each panel. Sharpened map after post processing in Relion is presented in a close-up view of the interface between A-site tRNA (blue), P-site tRNA (green) and their respective codons in mRNA (yellow). E-site tRNA is highlighted in dark magenta.

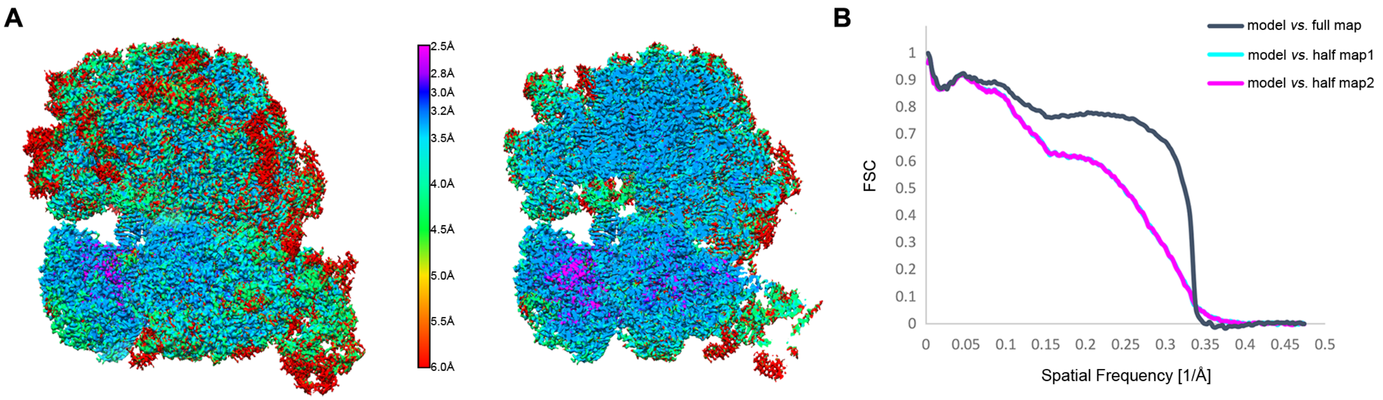

Supplementary Figure S2| Local resolution and FSC curves. (**A**) Surface (left) and cross-section (right) rendering of the *E. gracilis* density maps colored according to local resolution. (**B**) Evaluation of model map correlation. FSC curves of the final refined model *vs.* the final cryo-EM maps (full dataset, grey), of the outcome of model refinement with a half map vs. the same map (magenta), and of the outcome of model refinement with a half map vs. the other half map (cyan). The excellent agreement between magenta and cyan curves indicates lack of over-fitting.

A

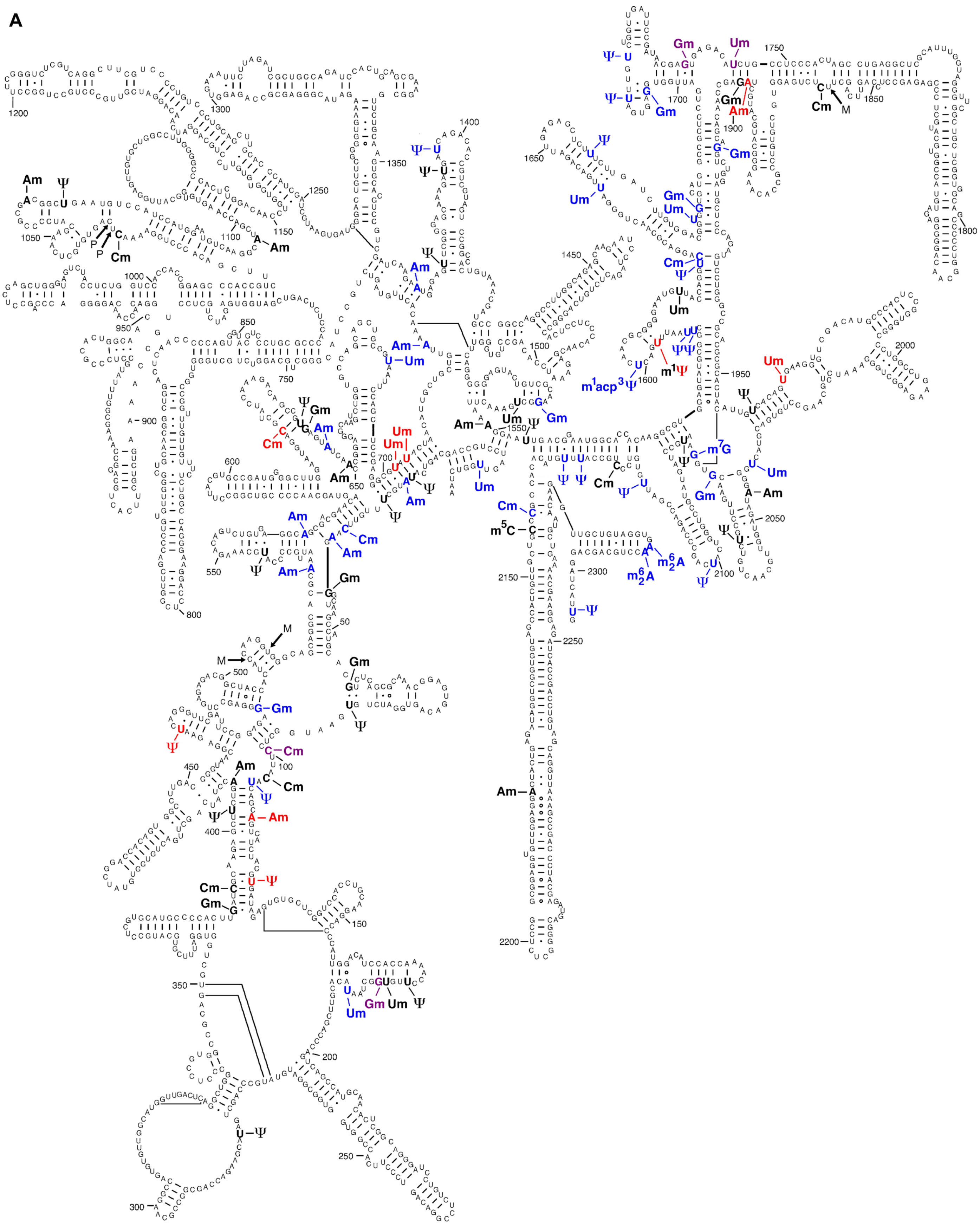

B

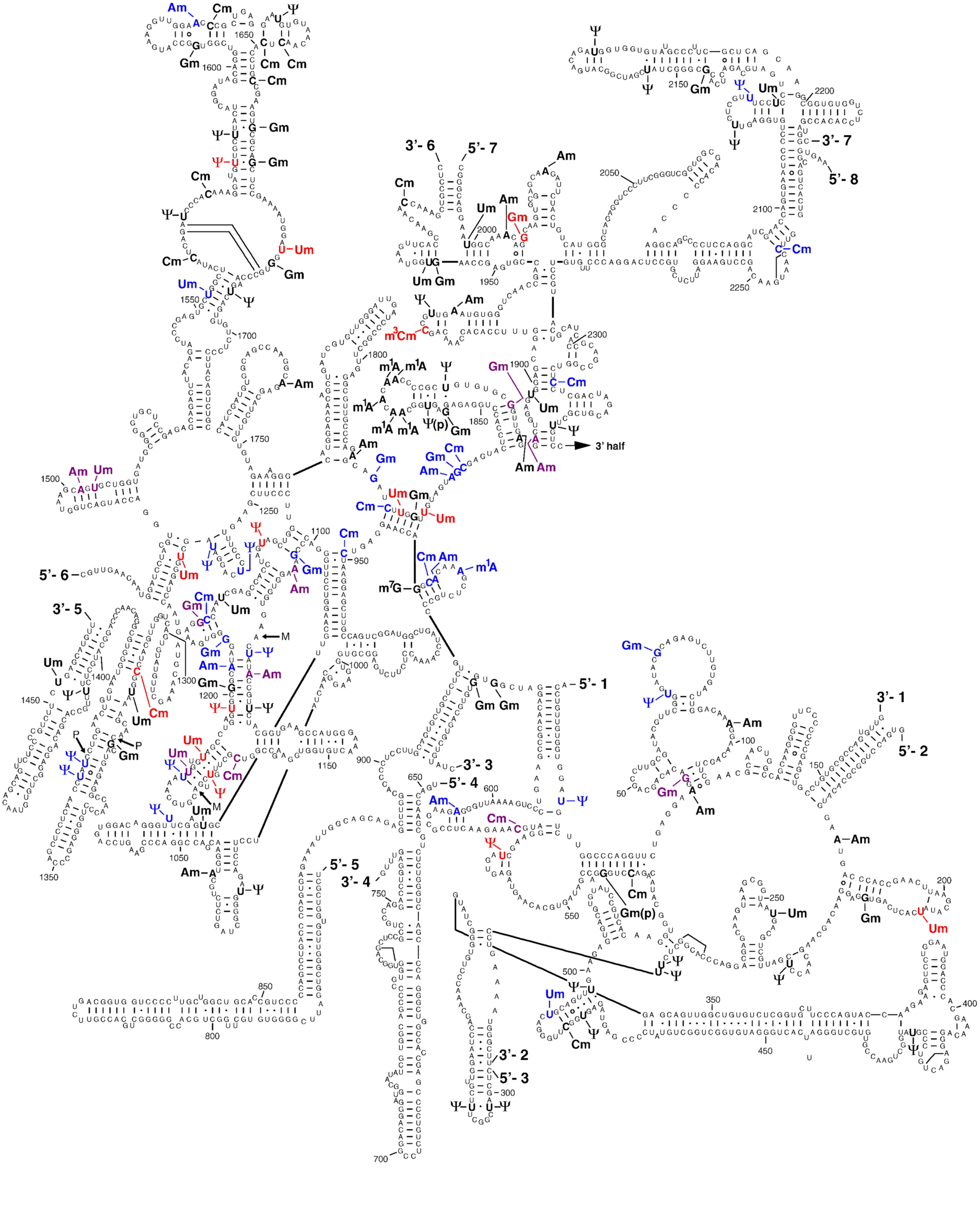

C

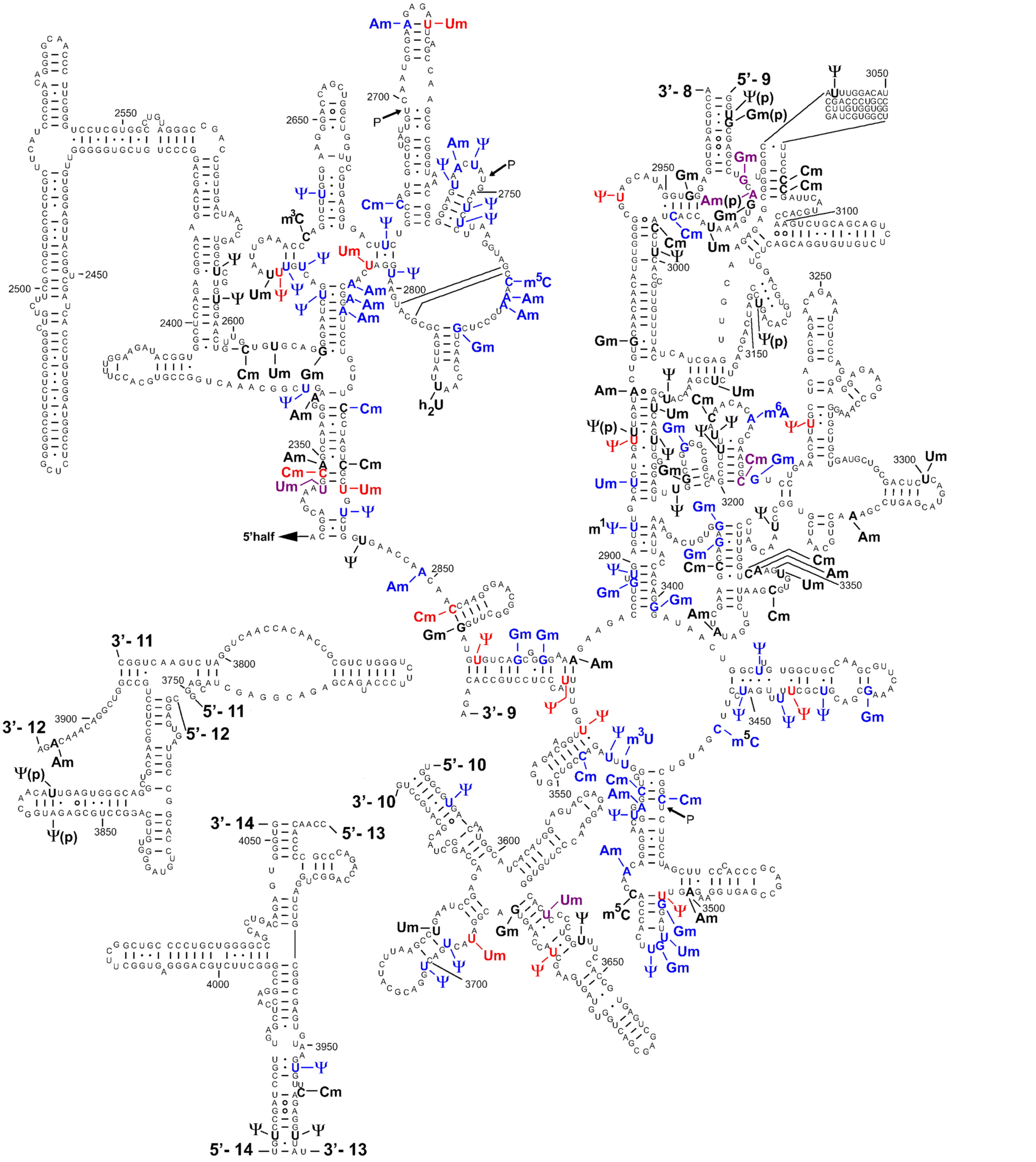

Supplementary Figure S3| Revised secondary structure diagrams for *E. gracilis* SSU rRNA (A), LSU rRNA 5'-half (B) and LSU rRNA 3'-half (C). Diagrams are adapted from the corresponding images previously published in Figure 1 in (1) and include the rRNA modifications detected in that study by chemical and enzymatic mapping and confirmed by MS and partially observed by cryo-EM in the present study (See **Supplementary Table S5** for more details). Secondary structure diagrams (1) have been revised to take account of alternate/additional base pairing (including proposed tertiary interactions) supported by the 3D cryo-EM structure determined here. Modified sites are displayed as enlarged fonts, with the identities of the modifications as indicated in (1). Three sites in LSU rRNA that were not definitively identified in (1) were elucidated in the present study and have been revised accordingly in the corresponding diagrams. These include m^{3}Cm1923 and m^{3}C2636; C2794, previously suspected to be Cm, was found to be an unmodified cytidine. Modifications are color-coded according to the following criteria: black, *Euglena*-specific; blue, present in at least one other eukaryote with none having a different modification; purple, a different Nm in at least one other eukaryote; red, the other type of modification (Nm *versus* Ψ) is present in at least one other eukaryote. The positions of highly conserved modifications in other eukaryotes that are not modified in *Euglena* rRNA are indicated by small arrows labeled as M (Nm) or P (Ψ).

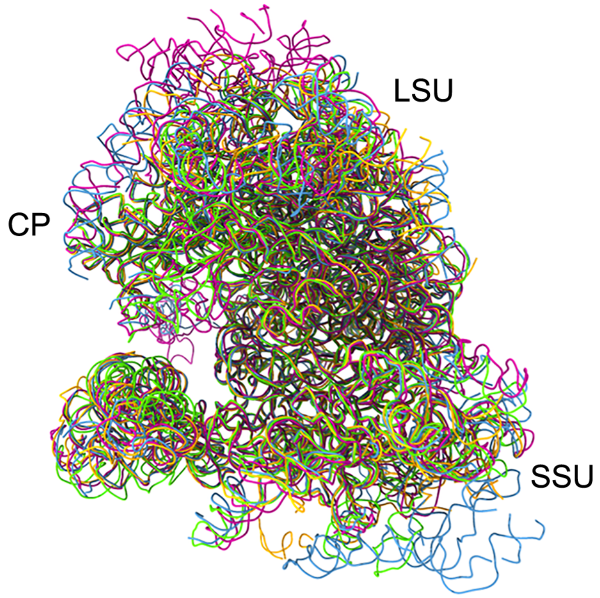

Supplementary Figure S4| *Euglena* rRNA compared with that of other eukaryotes. *Euglena* LSU RNA is fragmented into 14 discrete RNA chains. Nevertheless, the overall rRNA assembly resembles the one in other eukaryotes. Comparison with human (magenta - PDB ID 4UG0), yeast (green - PDB ID 4V88) and *Leishmania* (yellow - PDB ID 6AZ3) rRNA. *Euglena* rRNA is in blue.

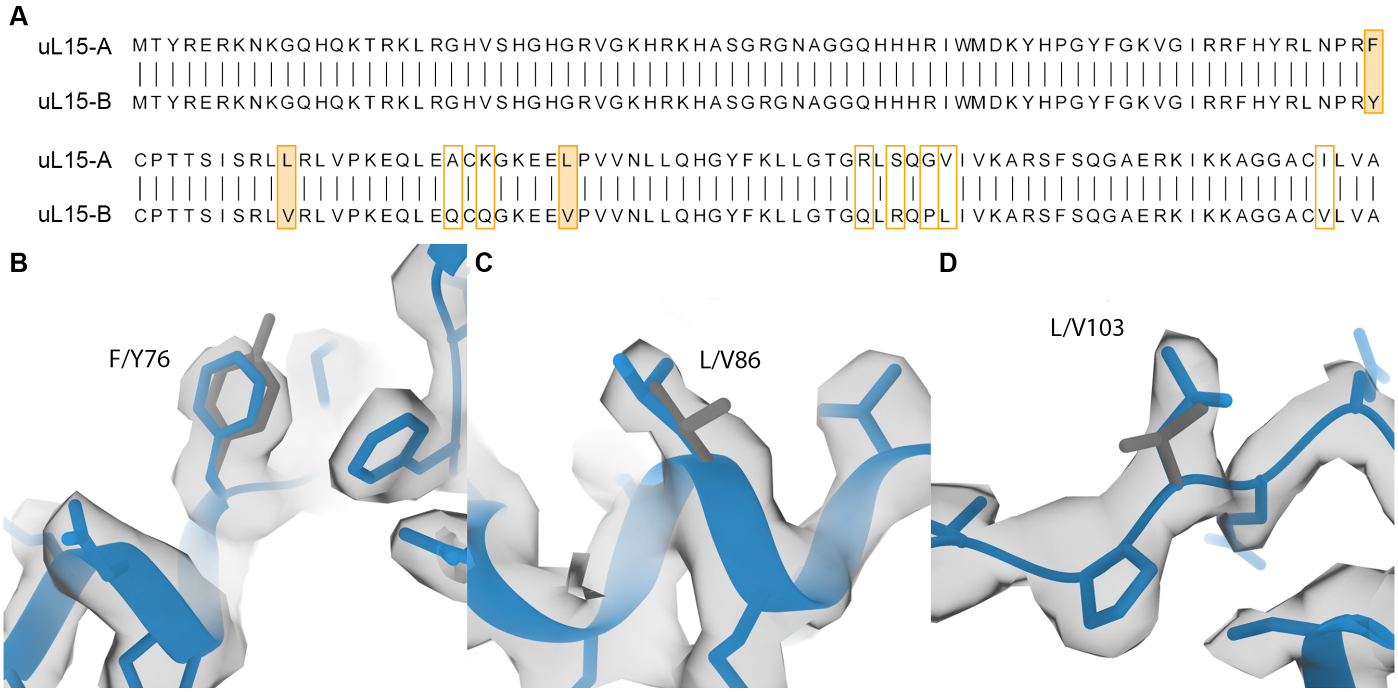

Supplementary Figure S5| uL15 has two paralogs in *Euglena*. (**A**) A transcriptome analysis revealed two paralogs for uL15. The paralogs share 93% amino acid sequence identity. Visualization of protein side chains with indicated features (shaded in orange) in the density map demonstrated that paralog A is localized within the purified ribosome population. Snapshots of side chains in densities are in (**B-D**).

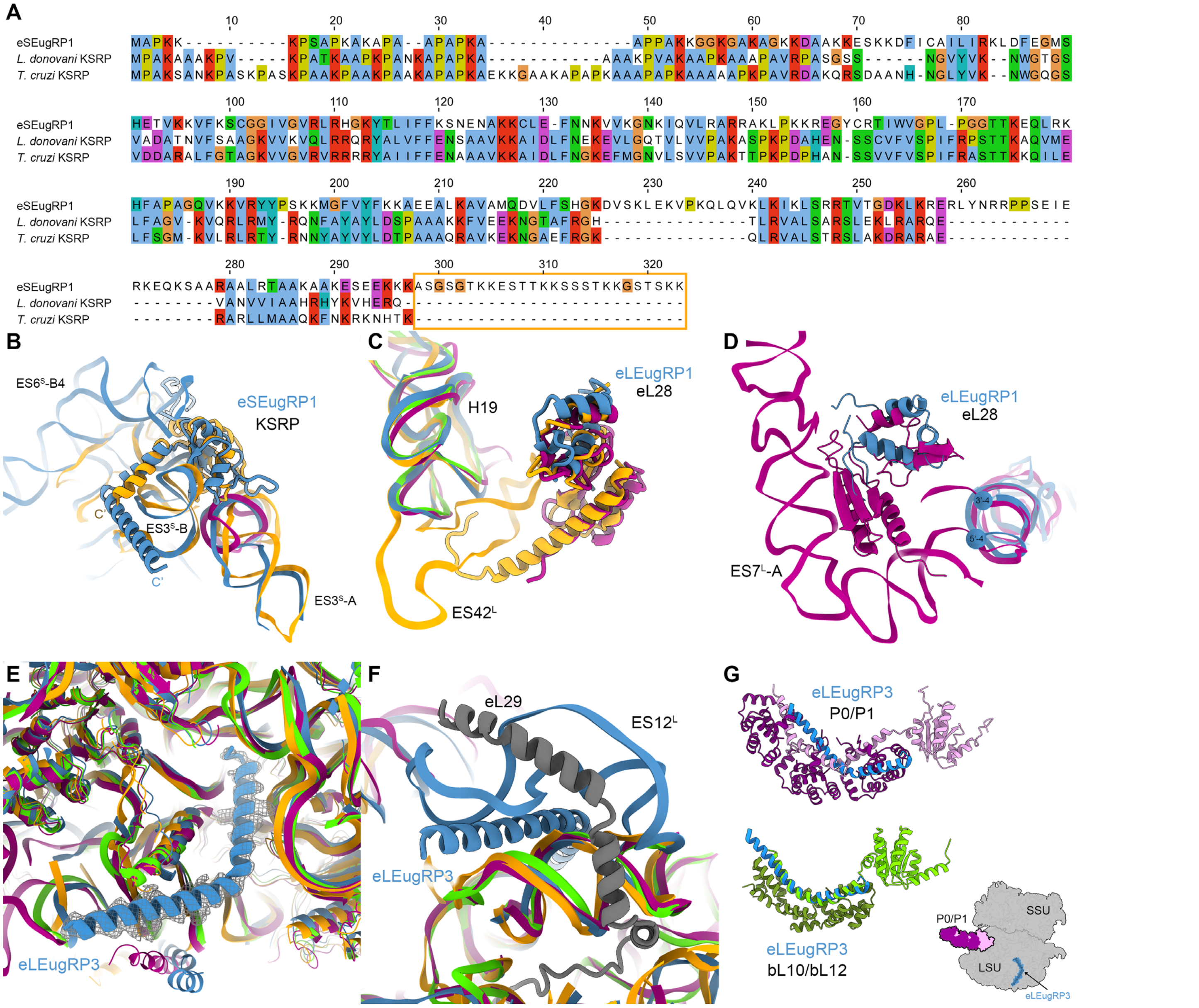

Supplementary Figure S6| Analysis of *Euglena*-specific RPs. (**A**) Sequence alignment of eSEug1 with its *Leishmania* and *Trypanosoma* KSRP homologs. *Euglena*-specific C-terminus extension is indicated by an orange box. Structural comparison is in main **Figure 2C** and in **B**. (**B**) eSEug1 is localized within an ensemble of rRNA expansion segments in the SSU foot region (ES3^S and ES6^S). This segment is significantly enhanced in *Euglena* (ES6^S-B4, blue) compared to human (purple - PDB ID 4UG0) as well as compared to kinetoplastids (*Leishmania*, yellow - PDB ID 6AZ3). eSEug1 also encompasses an additional C-terminal helix compared to its kinetoplastid homologs. This extension maintains further contacts with ES3^{S}b, which is clearly visualized in the *Euglena* structure compared to previously reported structures of eukaryotic ribosomes in which this region has been excluded due to high mobility (e.g. PDBs 4UG0, 6EK0, 6OLF, 5AJ0). (**C**) eL28 is a LSU RP that is highly conserved in eukaryotes but is missing in *Euglena*. eLEgr1 is localized in close proximity to H19 of LSU and although no sequence or structural similarity is evident between this *Euglena*-specific protein and eL28, the protein largely overlaps the eL28 position in other eukaryotes. eL28 is also missing in yeast ribosomes (green). In kinetoplastids (*Leishmania* - yellow), the C-terminal region of eL28 is further extended and stabilizes a unique kinetoplastid rRNA extension (ES42L) that is significantly shortened in the *Euglena* ribosome (blue). (**D**) Human eL28 (purple) is larger compared to eLEgr1 (blue) and maintains close contact with a human-specific ES7La. (**E**) Superposition of ribosomes derived from human (purple), yeast (green), *Leishmania* (yellow) and *Euglena* (blue) indicating that eLEgr3 is a *bona fide Euglena* protein. EM density is contoured around the protein. (**F**) eLEgr3 serves to stabilize an ES12^L that is considerably longer than in other ribosome species. eL29 (dark grey), which is highly conserved, serves to further stabilize this unique extension. Superposition of ribosomes derived from human, yeast, *Leishmania* and *Euglena* indicates that this ES is unique to the *Euglena* ribosome. Color scheme matches that in **E**. (**G**) eLEgr3 structurally resembles RP uL10 (PDB ID 1ZAV), localized to the L7/12 stalk in prokaryotes (green). This stalk is represented by the P0/P1 proteins in eukaryotes (purple), homologs of which exist in *Euglena*. However, eLEgr3 is localized to a remote region of the ribosome compared to this stalk. A figure indicating the localization of eLEgr3 compared to P0/P1 proteins is shown in the bottom right-hand corner of this panel. In **B-G**, *Euglena* RNA and proteins are in blue; yeast, human and kinetoplastid counterparts are in green, magenta and yellow, respectively.

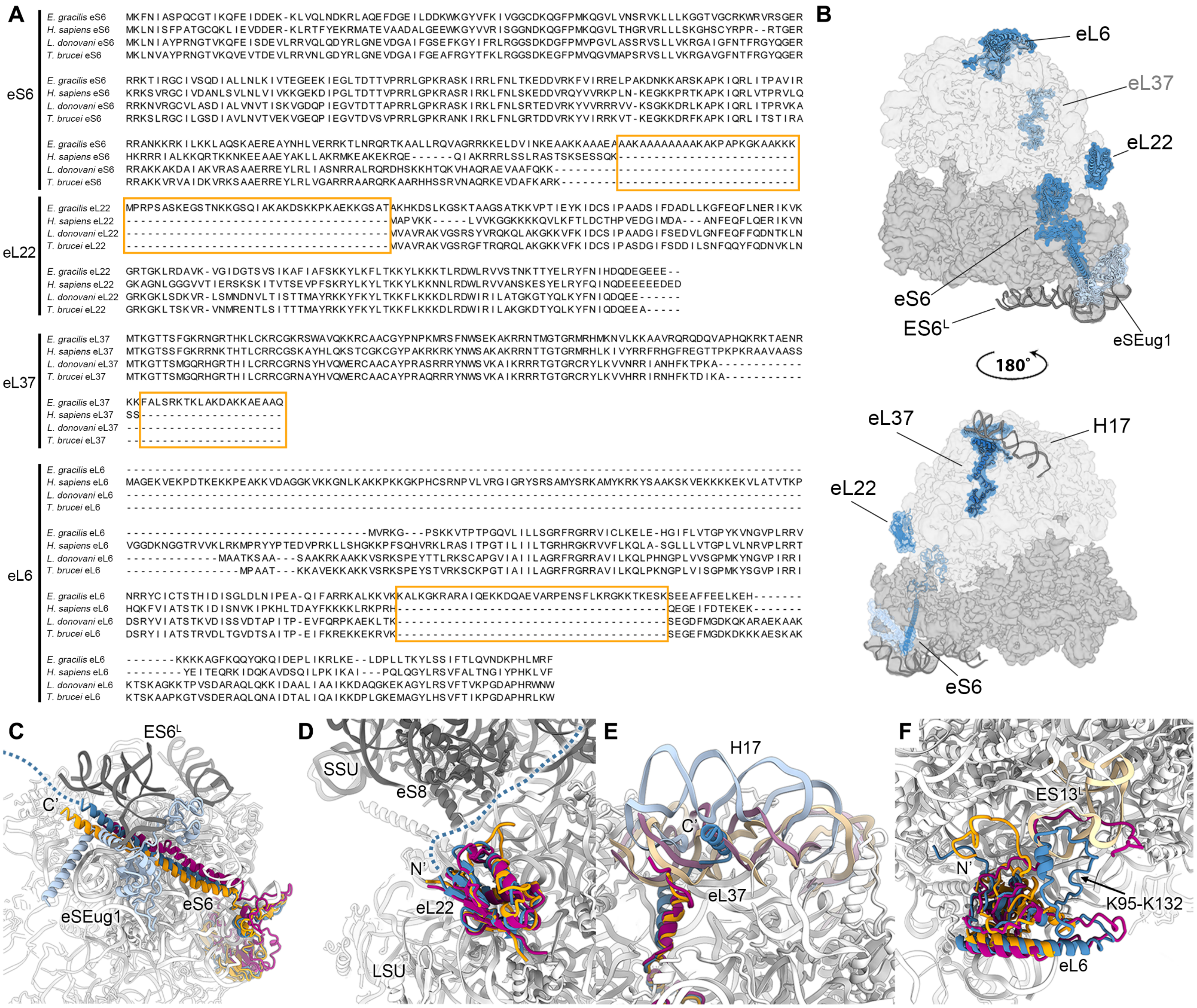

Supplementary Figure S7| Unique *E. gracilis* RP extensions and insertions. (**A**) Alignment of *Euglena* RPs with those of other eukaryotes highlighted four RPs that are significantly longer in *Euglena*. *Euglena*-specific extensions/insertions are indicated by an orange box. Structural implications are depicted below. (**B**) Localization of the RPs within the full ribosome context. (**C**) The C-terminal extension of eS6 is localized to ES6^{S}, which is significantly enlarged in *Euglena*. eSEug1, the homolog of KSRP, is in close proximity and maintains extensive interactions with its C-terminal region. (**D**) eL22 bears an extended N-terminus that might serve as a *Euglena*-specific ribosomal bridge. (**E**) eL37 has an extended C-terminus that adds an additional helical domain to the protein. This helix interacts with H17, pushing it 38 Å away compared to other reported ribosomes. (**F**) eL6 harbors an extended internal loop that occupies a ribosomal cavity that is utilized by an rRNA expansion segment in kinetoplastids. In panels **C-F**, *Euglena* proteins are in blue, human (PDB ID 4UG0) in purple, and *T. cruzi* (PDB IDs 5T5H and 5OPT for LSU and SSU, respectively) as representative kinetoplastid, in yellow. Protein extensions that were not modeled due to high residual mobility are indicated by a dashed line.

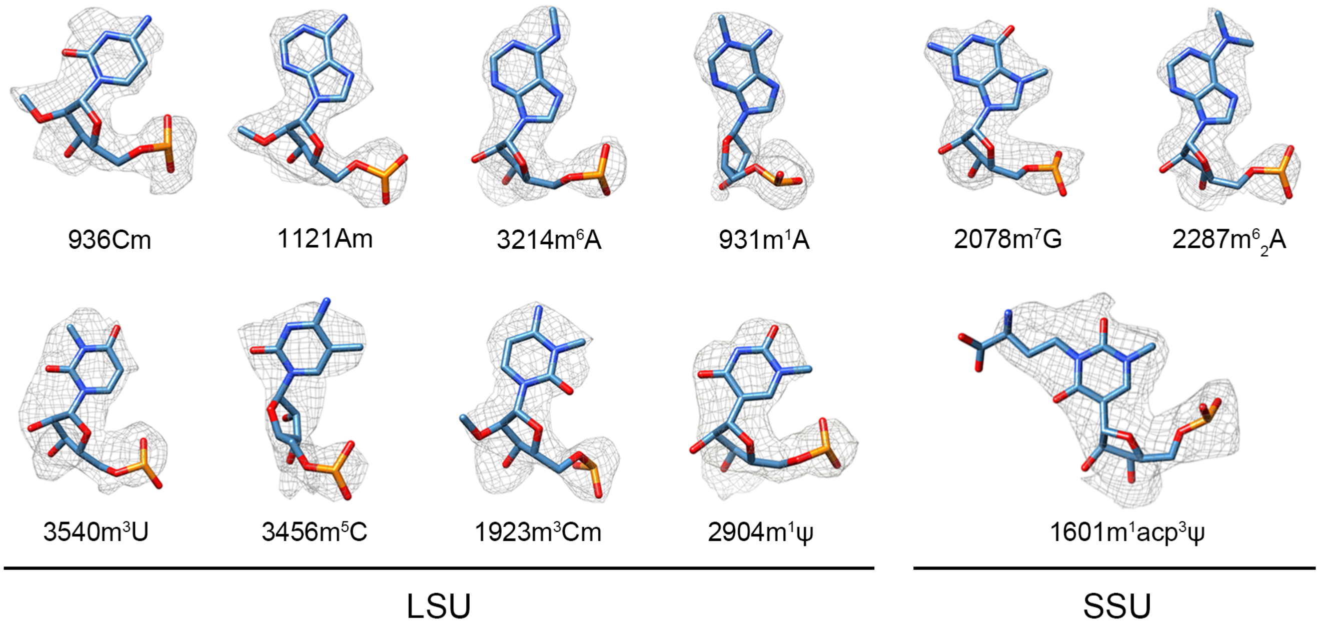

Supplementary Figure S8| *E. gracilis* rRNA modifications. Selected modifications mapped onto their corresponding densities as observed by our cryo-EM analysis.

Supplementary Table S1| Cryo-EM data collection and model refinement.

**Data collection**	
Microscope	Titan Krios
Camera	CMOS (Falcon II)
Voltage (kV)	300
Magnification	133K
Pixel size ($Å.px^{-1}$)	1.05
Defocus range (µm)	-1.5-(-3.5)
Total dose ($e/Å^2$)	35
Dose per frame	1.52
Micrographs collected	3,228
**Refinement**	
Number of particles (autopicked)	519675
Number of particles (used for 3D reconstruction)	176,308
Resolution (Å; at FSC[a] = 0.143)	3.15
CC[a] (model to map fit)	0.84 [b]
**RMS[a] deviation**	
Bonds (˚)	0.006
Angles (˚)	0.711
Chirality (˚)	0.041
Planarity (˚)	0.05
**Validation[c]**	
Clashscore[d]	12.15
**Proteins**	
MolProbity score	2.07
Rotamers outliers (%)	0.04
Ramachandran favored (%)	92.43
Ramachandran allowed (%)	7.56
Ramachandran outliers (%)	0.01
**RNA**	
Correct sugar pucker (%)	99.3
Correct backbone conformation	78.85

[a]FSC, Fourier shell correlation; CC, correlation coefficient; RMS, root-mean square.
[b]Only across atoms in the model; compiled using Phenix (2)
[c]Compiled using MolProbity (3).
[d]Clashscore is the number of serious steric overlaps (>0.4 Å) per 1000 atoms.

Supplementary Table S2| Protein and rRNA compositions.

Universal name	Chain ID	Length	Modeled range
**18S**	1	2306 b	1-2304
**A- tRNA**	2	76 b	1-76
**P- tRNA**	3	76 b	1-76
**E- tRNA**	4	76 b	1-76
**mRNA**	5	13 b	1-12
**eS1**	A	249 aa	20-240
**uS2**	B	261 aa	4-208
**uS3**	C	220 aa	5-216
**uS4**	D	196 aa	2-178
**eS4**	E	271 aa	2-266
**uS5**	F	257 aa	32-252
**eS6**	G	283 aa	1-248
**uS7**	H	190 aa	2-190
**eS7**	I	200 aa	10-200
**uS8**	J	130 aa	2-130
**eS8**	K	304 aa	2-303
**uS9**	L	151 aa	9-151
**uS10**	M	121 aa	20-120
**eS10**	N	152 aa	1-80
**uS11**	O	152 aa	18-152
**uS12**	P	143 aa	2-142
**eS12**	Q	139 aa	21-138
**uS13**	R	153 aa	2-144
**uS14**	S	55 aa	4-55
**uS15**	T	151 aa	2-150
**uS17**	U	164 aa	2-149
**eS17**	V	145 aa	2-126
**uS19**	W	150 aa	20-136
**eS19**	X	148 aa	5-147
**eS21**	Y	96 aa	1-82
**eS24**	Z	137 aa	6-132
**eS25**	a	119 aa	45-114
**eS26**	b	120 aa	2-98
**eS27**	c	86 aa	2-85
**eS28**	d	76 aa	13-75
**eS30**	e	67 aa	2-67
**eS31**	f	157 aa	91-144
**eSEug1**	g	296aa	50-262
**RACK1**	h	317 aa	7-316

Universal name	Chain ID	Length	Modeled range
**5.8S**	A	163b	1-163 (163)
**26S-I**	B	133 b	164-296(133)
**26S-II**	C	351 b	297-646(350)
**26S-III**	D	116 b	650-760(111)
**26S-IV**	E	698 b	764-1461(698)
**26S-V**	F	527 b	1464-1987(524)
**26S-VI**	G	235 b	1991-2222(232)
**26S-VII**	H	744 b	2224-2938(715)
**26S-VIII**	I	617 b	2997-3584(588)
**26S-IX**	J	164 b	3586-3748(163)
**26S-X**	K	65 b	3751-2812(62)
**26S-XI**	L	96 b	3813-3907(95)
**26S-XII**	M	58 b	3910-3965(56)
**26S-XIII**	N	86 b	3967-4052(86)
**5S**	O	120 b	1-120
**P0**	-	348 aa	-
**P1,P2**	-	112,111 aa	-
**uL1**	-	78 aa	-
**uL2**	P	264 aa	2-250
**uL3**	Q	410 aa	2-393
**uL4**	R	375 aa	3-367
**uL5**	S	191 aa	6-183
**uL6**	T	193 aa	1-190
**eL6**	U	195 aa	2-195
**eL8**	V	295 aa	58-291
**uL11**	-	165 aa	-
**uL13**	W	209 aa	2-209
**eL13**	X	226 aa	2-220
**uL14**	Y	140 aa	7-140
**eL14**	Z	219 aa	2-218
**uL15**	a	152 aa	2-152
**eL15**	b	204 aa	2-204
**uL16**	c	215 aa	1-214
**uL18**	d	260 aa	1-260
**eL18**	e	193 aa	2-193
**eL19**	f	250 aa	3-166
**eL20**	g	182 aa	2-182
**eL21**	h	159 aa	2-159
**uL22**	i	164 aa	3-157
**eL22**	j	170 aa	60-160
**uL23**	k	163 aa	33-163
**uL24**	l	146 aa	2-125
**eL24**	m	157 aa	1-66
**eL27**	n	134 aa	2-134
**eLEgr1**	o	72 aa	12-72
**uL29**	p	123 aa	2-122
**eL29**	q	117 aa	2-92
**uL30**	r	242 aa	25-242
**eL30**	s	109 aa	9-105
**eL31**	t	151 aa	41-151
**eL32**	u	139 aa	6-133
**eL33**	v	115 aa	2-115
**eL34**	w	128 aa	2-115
**eL36**	x	106 aa	2-103
**eL37**	y	117 aa	2-107
**eL38**	z	82 aa	2-71
**eL39**	1	51 aa	2-51
**eL40**	2	126 aa	75-125
**eL41**	3	34 aa	2-34
**eL42**	5	106 aa	2-98
**eL43**	4	92 aa	2-91
**eLEgr2**	6	69 aa	9-67
**eLEgr3**	7	64 aa	5-58

Supplementary Table S3| Cytoplasmic RP sequences in *E. gracilis*[*].

**RP name** (New nomenclature)	**RP name** (Old nomenclature)	**Sequence**
**SSU**		
**eS1**	S3A	MAVGKNKSVKKGQAKRKIVDFMTRKEWYDVVAPTTFTKRSICKTLVNKSVGNKNCTDNLKGRVFELNLGDLNEDESQAHRNIRLRVDDVVGRNCLTNFHGMSLTTDKLRSLVRKWCTLVEASMDLKTSDGFLLRIFVIGFTKRRPNQVRKNCYAQHSQVLRLRKKMFEIISAAVTKSDLQSCVKKFQLETIGKDIETASSRYYPLRDVHVRKVKVLHLPKFEPNKLLQEVHGGELPKSWEEGPTGAAVE
**uS2**	SA	MSKGRPILDPTEDDIRKLLACKVHLGTKNLVKQMERYVYARRKDGIHVLDLHMTWQKLVLAARVLVAIENPADVCAISARPYGQRAILKFSQYTGAQYVASRFTPGALTNQIQDTFMQPRILLITDPRTDHQAITESAYGNVPVIAFCDTDSPLVYVDLAIPCNNRGQKAIGMLYWMLTREILRMRGTCPRAVPWEIKVDLFFYRDPEEVLKKEEEVQQIAAPVADLYTGAQAQVQVDTGAAANWGDETGTWEAGGGWGAE
**uS3**	S3	MANTGNLSKKRKFVADGVFYAELNEFLKRELAEDGYSGVEVRVTPQRTEIIIRATRTKEVLGEKGRRIRELTSVVQKRFNFPDNGVELYAERVANRGLSAIAQAESLRYKLMGGLAVRRACYGVLRFVMDNQAKGCEIIVSGKLRAQRAKSMKFKDGYMIKSGHPAEIYVDKAVRHVYLRQGVLGIKVSIMLPFDPTGKMGPDRPIPDQVTIINPKDEPL
**eS4**	S4	MARGPKKHLKRLAAPKHWMMDKLGGIFAPKPRAGPHKSQQCLPLILIVRNRLKYALNYREAHMILKQRYVKVDGKVRTDIKFPAGFMDVIEIPKTGDKFRVLYDTKGRFILHKIKGEEVDFKLCKVVKKGTAKKETPYVVTHDGRTLRFPDPNLKKNDTVVIDLKTGKIKEWVRFKVGCLLMVTSGANTGRVGELVNRERHPGSFDIIHLKDAADNKFATRADNVFVIGSSIYHPLVSLPKLKGVKLSIVEDREKKLAHNSKKQKQKKQKR
**uS4**	S9	MVRKYRNFSKVYSTPRRPFDKERLDKELKLCGEYGLRCKKEVWRVGAVLTKMRSAARQLLTLEEGHLRRQIEGAALLRRCQKYGLLDESKLKLDYVLSLTVPDFLERRLQTIVFKLGLAKSIHHARVLIAQRHIRVGKQICTSPSFLVRMDSEKHIDFAPQSPLGGGPPGRVKRKKLAAESKKKDGGDDGGDEEED
**uS5**	S2	MSGEKGGKGFGGKGKGKGKGKGKGKGKGKIEEKEKEWIPVTKLGRLVKEAKLRKLEEIYLFSLPVKEYQIVDEFLPSPGKLKDEVMKISPVQKQTKAGQRTRFKAYVVVGDCDGHVGLGVRCSKEVATAIRASIQAAKVAVIPVRRGYWGNKIGLPHTVPCKVTGKCGSCRVRLVPAPRGAGIVAAGVPKKLLQFAGVEDCFTQSIGQTRTLGNFVKATFAALSKTYGFLTPDLWPETHFVKDPYQEHTDFLSQGLKMVKA
**eS6**	S6	MKFNIASPQCGTIKQFEIDDEKKLVQLNDKRLAQEFDGEILDDKWKGYVFKIVGGCDKQGFPMKQGVLVNSRVKLLLKGGTVGCRKWRVRSGERRRKTIRGCIVSQDIALLNLKIVTEGEEKIEGLTDTTVPRRLGPKRASKIRRLFNLTKEDDVRKFVIRRELPAKDNKKARSKAPKIQRLITPAVIRRRANKKRKILKKLAQSKAEREAYNHLVERRKTLNRQRTKAALLRQVAGRRKKELDVINKEAAKKAAAEAAAKAAAAAAAAKAKPAPKGKAAKKK
**eS7**	S7	MATTQVVGPRKKLRKSARKSATPLEDEVAQAIFDLEVNNKSLKPVLQPLYVNTAKEVDIGHGKRAVVIFSPLRFLKKFHRIHKQLTAELEKKFSGKQVLVIGQRKITRQGKHNKNKIPRTRTAVSVRENILADLLYPVDVVGRRWRHKTDGSKQTKIFLDAREKDKVEGKIEAISVVYKKLTGVDASFGFMTNPLLQQFQ
**uS7**	S5	MSKTTPLLFKKWSSAEVDVNDLSLQDYISKRSVYLPHTAQRWTKKRFRKAQCPITERLVNSLMYHGRNNGKKAMAVRHLKACFEIISLVTDQNPIQVLVDAITKAGPREDSTRVGTAGVVRRQSVDVSPLRRVNQAIYLITTGAREASFRTIKTFPECLADEIINASKGSSNSYAIKKKDEIERVAKANR
**eS8**	S8	MGITRWDLHKKRATGGKRHIHRKKRKFELGRPPAATKLVHGERRVRRVRTRGGNRKWRALRLSGGIFSWGSENTAKHCRILDVVYNATNNELVRTKTLVKGCIVQIDGTPFRNWYYKWYGIRLCNPKEVEKERRKQAGIAAKKKQSRKKVDEEEEEVKKVSKKDKKGSKGKEAKKSAKKDEEEEKPSKKEKKGKADKKADDKKDSKKKEKAETAEKKTAEKKEGEKKKGKRVYKNHLRKTHLRRKQKGFGLEEGLQAQLAVPVCKILARISSRPGQDGRANGYILEGKELEFYQKKLDKKKHKK
**uS8**	S15A	MVRQSTLADCLKCISNAEKRGKRQVLVRPCSKTILRFLRVMQKHGYIGDFEVVDNHRGNKIVIELNGRLNKCGVISPRYDISLPQMEKWITSLLPSRQFGFIVLTTSHGIMDHEEARKKHTGGKILGFFY
**uS9**	S16	MADRKKLAESKKQKVMTFGKKKHAIAVVTCNKGKGLVKCNGVPIDLLQPEPLRLKVMEPILIAGKHRFRNVDMRIRVRGGGQTSQLYAVRQAIAKALVAYYQKYVDEAQKLEIKDAYLQYDRTLLVADPRRTEPKKFGGHGARARRTKSYR
**eS10**	S10	MLVSKKNREEVYQYLFKEGVLVAKKDPGSKHPKMDVQNLVVIELMRGFASKGFVKEQYAWRHYYWYLTNEGIEYLRQYLHLPAEIVPSTLKKSTRAPAQRAVEGDRPPRMGRGGGGYGGGRDEYRKAQPAPGEFNPTFGGGRGLGRGAAPAE
**uS10**	S20	MAGIPPVKKEAPAAAAAQIHKIRITLSSKNPKALEKVCAELIKGAKDHKLRVRGPVKIPTKVLRITTRKSPCGEGTNTWDRYQMRIHKRLIDLHSPSEVVKEITSINIEPGVEVEVTVADV
**uS11**	S14	MPGKKKEVVEKVPAVQRYGPQTAAGDLVFGVCHIYASFNDTFVHVTDISGRETISRVTGGMKVKADRDESSPYAAMLAAQDVAERCKECGITALHIKMRATGGTRSKQPGPGAQSALRALARSGMKIGRIEDVTPIPTDSTRRKGGRRGRRL
**eS12**	S12	MSDTEETPEVQPQAEDDMPSDLMSALKEVLKRARAHDGLAIGLHAACKALDKRAAHLCVLAEDCTEAAYTRLVEALCQSHGIDLIKVPERLKLGEWVGLGSYDSAGNVKKMVACSCAVVKDYGESSKALTMVLEHFQRS
**uS12**	S23	MGKPHGIRTARKLRSHRREQRWADKDYNKRNLGTTLKCSPFGGTSHVKGIVLEKVGVEAKQPNSAIRKCVRVQLIKNGKKICAFVPKDGCLNFIEENDEVLVSGFGRSGHAVGDLPGIRFKVVKVSGVGLMALFREKKEKPRS
**uS13**	S18	MSLILQPDDFQHILRVLNTNIDGKNKVAYALTSIKGIGRRFAILCCKKADIDLNRRAGTLSNDEIARLVAIMQNPTQFKIPEWFLNRKKDPKDGKFSQSVSNVVEQKMRDDLEKLKKVRCHRGLRHMWGLRVRGQHTKTTGRRGRTVGVSKKK
**uS14**	S29	MGFKDIWRSHPGRGRGTRRCRISGNRHGIIRKYGLMMTRREFREIAGDIGFVKYN
**uS15**	S13	MGRMHSKGKGISNSCLPYRRTAPSWLKTSSREVVDQICKLAKKGVPPAQIGIQLRDSHGIGSVKSITGRKILRVLKHNGLAPEIPEDLYHMIKKAVNVRKHLEKSRKDKDAKFRLVLVESRIHRLARYYKQTKQLPATWKYESSTASALVA

**eS17**	S17	MGRVRTKTVKRASKVIIEKYFQKLTQDFQTNKKVICEIAIVPSTRLRNKIAGFTTKLMKRIARGPVRGISLKLQEEEREKRMDFAPEVSQVDQQIMDQGVEVDEDTLAMLAAMDMANISHLKKVQVQLPQATQQQQGGKGGKGRS
**uS17**	S11	MTEVELDDNAFHKQDNIFLAAKNPSSKWVAPGSKGRRRWWKKIGLGFKTPKAAILGTYIDHKCPFTSKVSIRGRILRGIIIRHKMFRTVVVRRNYLHYQKKYKRYEKRHKNWSVHISPAFDFSIGDEVVFGQCRPLSKTVTFNVLQVVPKSKKVAAQRKEFEKF
**eS19**	S19	MTGGKPAVVKDVDPQKFIAAYAAYLKKAGKIEIPKYVDVIKLAQFKELAPADPDWYYTRCAAIARRIYINPGTGVGGFRKVFGGNDRRGVRPGRFTKSSGGIIRHCLQQLEKMGIVEVASNGGRIMTKEGQRDLDRIAQQVLYPEMFE
**uS19**	S15	MDMDAEKYAELKKKRTFKKFTYRGIELEGLLDLSNEELAQILPKRARRKFSRGLKRKHMALLKKLRRAKKDAALGEKPPTIKTHLRNMVIIPEMIGSMIGVYNGKIFNAVEIKGEMVGHYLGEFSITYRPVKHGRPGIGATHSSRFIPLK
**eS21**	S21	MLNDEGINVDAYIPRKCSATNQLITAKDHASVQFNVGLIDENGAYIGEYKTIAFAGFLRRGAASDQALNRLMLAANILKEKGESEVVDVPKQRRGH
**eS24**	S24	MTDDKKPPFRVSFKNFMTNRLMQRKQFNIEIEHPGRSTVPKKEIQQKISQIFKIQEENTIVLFGFRTKFGGGRSTGHGMIYDNLSICKRYEPKYRLIRLGLRSKKEGSRKQKKEKKNRMKKYRGGKKAKGAVAAKKK
**eS25**	S25	MPPKKDAKKDPKKDAKGAKKGKDSKSSGGGKAKKKKWSKGKVREKLHNAVMWEKVIRDKLYSEVPKYKVITVSVISDRLKVNGSLAREALKVLHAEGLIKPVNVNANCRLYTRATAETA
**eS26**	S26	MTVKRRNHGRNKKNRGHVCGIRCSNCGRMTPKDKAVKRFIVRNMVETAAVRDIADASVYYTGYNLPKLYLKMQYCISCAIHGRIVRVRSVENRKIRSPPQKLRRGGGQQKPGGGGGGGAR
**eS27**	S27	MSTFKDSNLLFPPEASEKRKHKLKRLVQGQNSFFMDVKCPSCWAITIVFSHVAKVVVCSSCNTVLAQPTGGKAKLTEGCSFRKKPE
**eS28**	S28	MSGQAPPKESGDQPKLAQVIKIIGRTGSRGGVTQVRVKILDDNRNDRTIIRNVKGPCRVDDILALMETEREARRLR
**eS30**	S30	MGKVHGSLARAGKVKGQTPKVPKQEKKKEAVGRAKKRLLYNKRFISAPVGLGGKRAQPNKQPAGKLG
**eS31**	S27A	MQIFVKTLTGKTITLEVESNDTIDNVKAKIQDKEGIPPDQQRLIFAGKQLEDGRTLADYNIQKESTLHLVLRLRGGGGKKKKKKNFTRPKKKKHKKKKVPLAVLKFYKVDDSGEITRTRMECPHPDCGAGVFMAAHKDRQYCGKCGLTYVFQKEGEE
**eSEug1**		MAPKKKPSAPKAKAPAAPAPKAAPPAKKGGKGAKAGKKDAAKKESKKDFICAILIRKLDFEGMSHETVKKVFKSCGGIVGVRLRHGKYTLIFFKSNENAKKCLEFNNKVVKGNKIQVLRARRAKLPKKREGYCRTIWVGPLPGGTTKEQLRKHFAPAGQVKKVRYYPSKKMGFVYFKKAEEALKAVAMQDVLFSHGKDVSKLEKVPKQLQVKLKIKLSRRTVTGDKLKRERLYNRRPPSEIERKEQKSAARAALRTAAKAAKESEEKKKASGSGTKKESTTKKSSSTKKGSTSKK
**RACK1**	RACK1	MDSTGHFAYLGQLKGHNGWVTALATPADDSDWVLSASRDKTLLKWKIERDPSGFVTGIPQRSLLGHSGFVQDVQLSSDGQFALSASWDSTMRLWDLSTGTCTRKFTGHAKDVLTCAFSADNRQIVSGSRDNLMKVWNTLGECKYTINEDGHTDWVSCVRFNPNPAAPLIVSGGWDKVVKVWSLTNLKLLTNLLGHTGYLNTVCCSPDGSLCASGGKDGAAMLWDLNKGEHLYELEGGDIIHALCFSPCRYWLCAATESSIKIWDLESKSIVAELVPELPAMSKKAMKPECISLQWSADGSTLYSGYTDHTIRVWGVS

LSU

**uL1**	L10A	MSKINPNTLQAAIQTLLSESHTKTGKPLRKFPESVDLQVNLKNYDTQKDKRFSGTVKLPRVARPRFKVCIIADAAHADAAQKLQVPMKNVDDLKKLNKNKKLVKKLCNEFDAFLASDSLIKQIPRIVGPHMNRAGKFPTSLSSGDDIMGRITEIRSTIKFQLKKVLCMGCCVGHVEMSAEDLKLNITLAINFLVSLLKKNWQNLKSVHIKSTMGKPQRVF
**uL2**	L8	MGKPVRGQRKGAGSIYITHGHKRQGQARLRPLDYGERKGFIRGVVKEIVHDSGRGAPLAKVQFRHPYKFKKVTHLMVACEGMYVGQYIYCGKKAQLAIGNVLPLSKMPEGTIVCNLEAKVADRSRLAKASGAYAIVISHNPDTGKSRVKLPSGQKKTVSSQCRAMIGLIAGGGRIEKPVLKAGNNYYRFKVKRNCWPRVRGVAMSPVDHPHGGGNHQHIGFPCTVSRNAPPGQKVGLIAARRTGRLRGGVKSGKDTEADSKKKK
**uL3**	L3	MSHRKFERPRHGNMGFLPRKRCRRERGRIKTFPKDDPSQAPHLTAFLCYKAGMTHVVRELDRPGSKMHKKEVVEAVSVMEAPPMIVVGLVGYAKTPRGLRCLKTVWAQHLPEQFKRCFYKNWSRSKKKAFSHYSANLMGPEGKKAYEAGIAKIKKFACVVRLIAIGQVKLLRIGQKKAHCMEIQINGGSIADKVEFGLKLFESSIPVDSVFKESEVVDCIGVTRGHGFEGVIHRWGVTRLPRKTHKGLRKVACIGAWHPARVGYTVPRAGQNGFHHRVEANKKIYKIGKSALVDKANARCETDLTDKTITPLGGFVRYGIVREDYLLIKGSVPGPIKRVMTIRKALRIKTNKAFTEEVQVKFIDTSSKFGHGKFQTREEKRKFMGPTKKSALRGVRKGKTGKEGKKSSKK
**uL4**	L4	MAARPLVTVQKEGKTVPLPKVFSSALRYDVVRFVHTNIAKNTRIPYAVSRRAGHKHSAESWGTGRAVARIPRISGGGTHRSGQGAFGNMCRGGRMFAPTKVFRKWHAKVPRKVRRLAVRAALAASAIPALVMARGHNLEDVPEIPLVVPNSVEALEKTKDAVKLLKDIGAYHDVEKVAATKKVRVGKGKRRNRRYCVRKGPLVVLSKRCEAQRAFRNLPGVDINFVSALNLLRLAPGGHIGRLIVFTEDAFNQLDKVLKVDTIEGLPDSKRVINSEEVQGVIRHKIKASRRKPQKNSSRPRLNPAHKVETKVQKKLYIERCKVNADPELRKAKEKKQKAAKKLWLLKVTGKVDKKGNPRKSTKTPKQDKKKKSKK
**uL5**	L11	MPEGENKASKRLKKLQNPMREIRVAKLIINICVGESGDRLTRAAKVVEQLTGQTPVLSKARLTIRSFNIRRNEKIAVHCTVRGQKAEELLERGLKVKEFELKKKNFSKNGNFGFGINEHIDLGIKYDPNTGIYGMDFYVVLERRGFRVARKKRKSGKVGKAHKITKKESIKWFIEKFDGIVTDKKKTTTDE
**eL6**	L6	MVRKGPSKKVTPTPGQVLILLSGRFRGRRVICLKELEHGIFLVTGPYKVNGVPLRRVNRRYCICTSTHIDISGLDLNIPEAQIFARRKALKKVKKALKGKRARAIQEKKDQAEVARPENSFLKRGKKTKESKSEEAFFEELKEHKKKKAGFKQQYQKQIDEPLIKRLKELDPLLTKYLSSIFTLQVNDKPHLMRF
**uL6**	L9	MRAIYAVHKTPIPEGVKVFVRNRRIVVRGPRGTLKREMRHVKYDMSVKGKKNKEFRCEVWFGKNQDEACIKTICSHVANMITGVTKGFRYKMRFAYAHFPINVSISDDGKAVEIRNFLGEKLIRKVEVREGVSASRTDPAKQKDELVLEGNDIEKVAQCAADIHQSCLVKNKDIRKFLDGIYVSSKETVVSEA

**eL8**	L7A	MGPKKAAAQPAASAKKAAPKVAPKKGSKSKDATGKKPAGKKGKDGKGKKAEAEVKKAGKPVQLFESRPKNFSVGQDLQPKRDLSRFVRWPAYIKRQRQKRILLKRLRVPPAINQFNHTVDRHLKKELFKFALKYKPESSFERRSRLKKEAEAKLKDPKAPASVPGPRVYSGAQRVFRLVEQKRAKLVLIAHDVDPIEIVLCLPALCRKQGIPWCIVKGKANLGKLVGLKTATSLAFVDIKNGDKTDFEKLTQSVKLAYNDKYEELSRKWGGLRLSKKSKQKMAKKKRIAAANAAK
**uL11**	L12	MPPKFDPNETKIIYVRATGGEMGATSTLAPKVGPLGLNAKKVGEDIMKASKEWKGLRVTVKLTIKNRQATAEIVPSSTSLVLRALKEPPRDRKKVKHIKHDGNITLEEIIDIARIMRPRSMARELKGTVKEILGTAFSVGCTVEGRNPKDVCDDVGAGIIEIPEK
**eL13**	L13	MVKKGNAPVPNAHFHKHWNPTGSQKGHVRCFFKQGPQKKIRRARRQKKAAVFPRPVKGPLRPIVHCKSQRFNMKTRLGRGFTTLELKTAGLTDRRYARTIGISVDRRRRNHCEESLALNAERIKTYLSKLVLFPLKAGKKLPEGVTEEDIKNPVQSKQDGFPKASSYGKNALKPEAPRKLTDQEKKRLTFQFLRKVQRDTKMVGKRIKRKAKKEAKAAKEAAKQK
**uL13**	L13A	MVKPKDRKWRGIVVDGKNHLMGRLAAVVAKQLLRGESICVVRCEDINISQSFMRNKFRLKNIMRKRHLTQPKRGPFHYRSPRKMFEKVVRSMLPYRTAHGTAAFERLRVAEGIPQPFAKKKRCICPAAHRILRLAPNRKFCRLGDLCTDIGWRHDKTIKDLEGKRKKLAELRWKKKKELTKLRRAAERLVDRIIPRIEYGDPKIKIGSL
**eL14**	L14	MVKAKRNFVEIGRVIYVRFGKNRDTLAVITDIIDHNWIFADGRAFPNGMKRGAVNIKDIALTHMKLKMGHGLRHRHLKELVAKEDVLNKWKKTTVARRLEKQKNKLNSTDLERWRIQYNRRQRSSLIKVKFNKLKKKALQTARRLHGKKNPIPFADRKKPQKQAPKEGKVKKDDKGRKLKDSERRKIKKCRINYKSLAGWQLDVNPGPIAGSSLVSKVH
**uL14**	L23	MSKRASGKGTGNKFRISLALPVAAVMNCADNTGAKNLYIISVKGIKGRLNKLPAACVGDMVLATVKKGKPELRKKVMPAIVVRQRKHWRRKDGTFIYFEDNAGVICNPKGEMKGSGVTGPVAKEAADIWPRVASNAGTVV
**eL15**	L15	MGVYTYMQFVWNKKQSDVMRFTQRIRAWEYRHSHRMVRLPRPTRTDKARRLGYKRKPGYVIWRVRVRRGGRKRPVPKGICYGKPKTAGVNHLMNAKNMRVIAEGRVGKHCGGLRVLNSYWVNADAIYKYFEVILVDPMHKAIRSDPEINWICKPRQKHREARGVTSAGRKHRGLRHKGHRATKARPSVRANWKRRNLVKFWRYR
**uL15 var1**	L27A var1	MTYRERKNKGQHQKTRKLRGHVSHGHGRVGKHRKHASGRGNAGGQHHHRIWMDKYHPGYFGKVGIRRFHYRLNPRFCPTTSISRLLRLVPKEQLEACKGKEELPVVNLLQHGYFKLLGTGRLSQGVIVKARSFSQGAERKIKKAGGACILVA
**uL15 var2**	L27A var2	MTYRERKNKGQHQKTRKLRGHVSHGHGRVGKHRKHASGRGNAGGQHHHRIWMDKYHPGYFGKVGIRRFHYRLNPRYCPTTSISRLVRLVPKEQLEQCQGKEEVPVVNLLQHGYFKLLGTGQLRQPLIVKARSFSQGAERKIKKAGGACVLVA
**uL16**	L10	MGRRPAKCYRYCKNKAYPKSRYCRGVPEAKIRIYDCGMRKLNADTFPLTYHLISMEREQIGSEALEAARINANKYMIKNAGKEAYHIRVRVHPFHVCRINKMLSCAGADRLQQGMRGAFGKPNGLAARVRIGQPLMSIRVKPQHEAVVVAALKRASYKFAGRQVIAKSTMWGFTEIRSENYVKWKEEGKFRVDGVSTKILRNRGRLHRAKRVRAA
**eL18**	L18	MGIDKPKQYKNKDKRHPTATNPYIRVLVKIYRFLARRVATPFNKAVLKRLMKSRMWRPRISTSRIKLAMRKPDKKDHICVCVSHVIHDDRVLGLKPLKVCALKFSKGARAAIESAGGQCLTFDQLAMITPKGSKCVLIRGKVTRRKQYKSFGAPGVPGSHAVPKLGNRKSALRGRRHENARGRRKSRAFKVKA
**uL18**	L5	MFVKIVKNRSYFSRLQIKYRRRREGKTNYRKRRLLVAQDKNKYNTPKHRLCVRMSNKDITAQIIIAKIQGDVVLAAAYAHELPIHGAVVGLTNFAGAYATGLLLARRILTKLGLADKYVGVAEANGEEYHVEAQEGRRPFKAFMDTGLARVTTGARIFAVLKGVVDGGVNVPHSMKRFPGYNRDKGEMDSETLRERIFAGHVAEYQKMLIREEPEKYQEVYSQYIAKGVKPGEVEDMWANCHASIRANPMAKLLSETFEV
**eL19**	L19	MVAGLKLQGRLAAALLKCGRNRVWLDPNETSDIAMANSRANVRKLIKDGFIIRKPVAVQSRARWRKLRAAKLKGRHTGPGKRRGTANARMPTKVLWIQRQRVLRRMLMRYRDAKKIDKHLYRELYMKCKGNVFKNKRLLMEHIHKAKAAKQKEKLIKDQLDAKKQKSLAKREKVKAAEVKRREREKEKVHDEKEKVKKDKEEKKAPAKEEKKAPAKEEKKAPAKEEKKAAKEEKKAPAKEEKKASKPGKK
**eL20**	L18A	MPGFSGIRHYEIVGRAYPTEKEPSPKVYKMTVFAKNSVVGKARFWKLMRKQNKVKKTHGQVLQIRRIFEKNPNTIKNYSILLRYQSKTGVMNVSKEYRDTTLCGAVHQMYMDMAGRHHARYLDIDIIGTTVLKPSQCLRPHIRQFLQHKVKFPLLHRITKKLPQHKATFLYSKPSTYRSGFM
**eL21**	L21	MPHSWGKKARTRDLFSKGFRCHGRPSLSTYLAVFKIGDYVDVVTDPSVQKGQPSKVFQGKTGTVWNVTPRAVGVLVNKRVRGRIVRKKISVRTEHVRKSRCQQDFKERVRHNELVKRTGVGKKIKRQPVGPREGQLLKLRKKPIPLGAKKFVFSEVYNF
**eL22**	L22	MPRPSASKEGSTNKKGSQIAKAKDSKKPKAEKKGSATAKHKDSLKGSKTAAGSATKKVPTIEYKIDCSIPAADSIFDADLLKGFEQFLNERIKVKGRTGKLRDAVKVGIDGTSVSIKAFIAFSKKYLKFLTKKYLKKKTLRDWLRVVSTNKTTYELRYFNIHDQDEGEEE
**uL22**	L17	MGKAKYSYTPKAEAKCAKARGTDLRVHFKNTRETVKTLHGMTVKKAFAYLRDVLARKRCIPFRRYGTGCGRTPQAKEFKHTRGRWPVKSVEYVQNLLKNAVANANTKGLDPNAMFISHIQANRAQQQRRRTYRAHGRINPYMSNPCHLEVVLVTKEEPVKPPEP
**uL23**	L23A	MAPKKEAKAPPKKSAAKPKAKAKGAKGDEKKPVKKAVFKRYNKIRTTVQFHRPYTRRTKGQKKYVRRSGRSVSIAQKKDQFHILKFPLTTESAMKKIEDNNTLVFIVDIRANKNQIKTAVRKMYDIKAARVNTLIRPDGLKKAYVKLMPDNDALDIANKIGIL
**eL24**	L24	MRKENCLFSGLPVHPGHGKRFVPTLVQSTRPVLTFVTAKTRKLYLRKKNPRVIRWTVTYRKLNKKTATVEEIRKRNKKSKKILRPIAGADLETIRQKKAQRDTIREASREAALKELEARKAKRVEAKTGGKDVKKAAPKSKPVAPKMPKTSVKKGGR
**uL24**	L26	MVNKVGKSGDRRKARKSYFTAPSHVRRVIMSARLSKDLRQKYKVKSLPIRKEDEVKVKRGSHKGRDGKVIACYRLKYAVHIDKITREKANGQTVQIGIHPSNVEITKLKLDKDRKKLLETKGRRKNKDSKDKTKVGQAEVAMQDVD
**eL27**	L27	MVKFLKAGRVVIVLQGRMAGKKAVIVQNSDTGNKERPYGHCVVAGIEQTPKKVTRRMSKKKIARRTKLKPFIKVVNHRHLMPTRYNIDLGAEFKGKISITDPTKRTASKRNVKKVFQQRYLAAKNKWFFQKLRF

**eL29**	L29	MAKSKNHTNHNQNYKDHRHPIRLKGRRNQISSKGLEQKLKKNRKKSRKGFLTPDVQKEIREKRSKKGEARR LKSYEKFKAKLPIWKAKHKAAVKAEKEQG(I/V)PDPKKASKQKEAAVKK
**uL29**	L35	MPKVKAHEIRQLEKKDLLKQLDDLKTELAQLRVAKQTSGAASKLCKIKIVRRSIARVLTVLNMKEKNTLRKLYK NKKYKPLDLRPKKTKKERLALSIIDRRRKTARQKKILHAYPMRKYYVKV
**eL30**	L30	MVSKKAKKQDQNINSRLQLVVKSGKYCLGYQSTLKSLRQGKSKLVIIANNCSPMRKSEVEYYAMLSKTSVHH YAGNNIDLGTACGKFFTASMLSIIDAGDSDIIRAMPQ
**uL30**	L7	MPRMTAVPPPERKKRVLADKKEIQEARIARKKAAKKKTVDKRKEMVRRYYRYRRLLNAQKSMLQNQRRLAK TFGHYFVEPEPKIAFVIRIRGICDMPPKPKKILQLLRLRQIFNGVFVRLNAATVNMLRVVSPWITWGYPSHQVV RNLMYKRAFAHFAHKRLPITNQLIEKALGKYKIICMEDLIYQLYTCGPRFREVSKFLWPFKLSAPRGGLKSKKK HFIEGGDSGNREIYINQLIRKMI
**eL31**	L31	MVKAEEKVQKKPVLKKDGKKVVAKKTKKSKPKPKVVKKTVKKAKNQPLSLETTINLHRVVFGCTFKKRRARKAI KAIRGFVRRVMKTHDVRIDPKLNKFVWSGGVKGVPFRARLRLDRKRSEDEDSKKKLYTVVSWVPVHDFKNL THKKIEE
**eL32**	L32	MARVGVNRTVTRKELKKKKHVPHFKRYQSWRHVRVSESWRKPHGIDSAFRRRFKGYPAMVGIGYKQPKNV RHVHPDGKRQFLVHNLKDLEVLLMYNKKYAAVIGHSVGARKRKSIVTRAKELQIKVTNANARLRAEEHE
**eL33**	L35A	MPKPIVRLFQPAVMTGFKRSKRKQDPHVAILAIKNVATRKHTDFYLGKRCVYLYRGKKKVAGRKGSKVKTNM RRIWGRITRSHGKAGCVKAVFKPNLPGESIGRKVRIYLFPSSI
**eL34**	L34	MAIGGTRVRYRRHNTKNTRSNKIRTVRTPGSRITVLYRKKLPKGPHTPVSLGHKPIPGVKRLRSIQRKSAPKR HLTVSRAYGGCLTHDLVRERIIRAFLIEEQKIVKRVLKAQSKKKRKFRKRAKADE
**eL36**	L36	MGKFGALPSAKLRRCSAVTPLPKPFRARDRIKKPKKKLRAVRDLVQEVVGFSPYERRMIELLKVGREKRALR FAKRRLGTHRQAKKKRDIMTEVLRKMRAAAHPKK
**eL37**	L37	MTKGTTSFGKRNGRTHKLCKRCGKRSWAVQKKRCAACGYPNPKMRSFNWSEKAKRRNTMGTGRMRHMK NVLKKAAVRQRQDQVAPHQKRKTAENRKKFALSRKTKLAKDAKKAEAAQ
**eL38**	L38	MPKEIRDIKQFLVICGRKDCKSVKIKKNVGNTKFKVRTKTYLYTLVVKDHKKSEKIQQSIPPNITKEVIGKGRRK VTKPAAK
**eL39**	L39	MGAIKTLKQKLKYGKKMKQNRPIPYWVRFKTNSKQRYNEKRRHWRRTKLKM
**eL40**	L40	MQVKVQTLVGRVLQIDLEEGASIDALKAKIEEEISLPSESQALFMDGKELDGESAEYKLDAATIHVVARLRGGV MMEPTLQALARKYNCEKMVCRKCYARLPLRSHNCRSKMCGHTSELRMKKKLK
**eL41**	L41	MGSISRPRGMRRKWRKKRMKRLQRRRRKMRQRSK
**eL42**	L36A	MVQYPKTRKTFCKGEPCKKHQTHKVVQYKVGKASVVAQGKRRYDMKQKGFGGQTKPVFHKKAKTTKKITL KLQCTSCKLIKQIKIKRCKHFELADRKGRSKGQPDW
**eL43**	L37A	MARRTVKVGITGKYGTRYGANLRKRVKKIEISKKAKHFCHWCGKFRFKRQAVGIWHCTSCGKTMAGGAWTL NTGNSTTVRSTIRRLREMTEQ
**eLEgr1**		MAEVELVSVPECKAQTVDKHVLWSCINFGTSNVALIDPYHPAHRGARKYINQFHSGKVPKTAAKAKEAKAEE
**eLEgr2**		MGGDDFEKKPLPDCLKELHEKQQAKLAKSKENYTPPKYNTPRKTTRERLNRRAQIKAALQRKKDKLKAE
**eLEgr3**		MPLKNNCFRRVYHSNWEYLLSLEKEADAEPKQKALRYKQEKKQQFREKGLKLAAAKTAEAAKSA
**P1**	LP1	MALQKNSELACTYASLILHDEGLEVTPDRISAMITAAGVQIESYYPSLFAKFLAGKNLGSMLSVSAGAAPAPAA AAAAPAAGGAAAPAAEAKKEEKKEEEEEDDDMGFGLFD
**P2**	LP2	MKHIAAYLLVALSGATPSKEKVIEVLKAGECEADEERLDSLISALEGKDINEVIAEGLSKIGSVSLGGGGGAAA PAAAAPAAAAAEAAPAKKEEKKEEEEEDEDMGFGLFD
**uL10**	LP0	MSGVEGKKKKVRPAKKKTQGGDLDRQFPTKKVEYFKRAHMFFDEYEKILVVLTDNVQSKQMQDIRISLRGK ALVMMGKNTTIKKILLDRLATGTEKNELLYQRLVREGLLAGNVGLIFTNGDLNTIKDIIDSNKIQAPARQGAVAP LDVIVPAGNTGLEPTKTSFFQALNINTKITKGTVEILKDELVLKAGDKVGSSEAALLQMLGIKPFFYGMAIVKIYD KGEVYDRRVLELTDDDIKKMFEGGIANVTGLSLGANITTEASLPHVMANAFKSVLAVTVGSDYVMESCNGKE LREAILSGKGLGGPAPAAAAAPAAAAAAAPAAAEAAPAKEEEEEDEDMGFGLFD

**Euglena*-specific proteins are highlighted in green.

**Red letters indicate alternative amino acid (I or V) at this position, as inferred from transcriptome data: likely a consequence of gene duplication and divergence. These alternative sequences were submitted as eL29a (V) and eL29b (I) and so have separate accession numbers.

Supplementary Table S4|Complete protein profile detected by MS.

Accession	Protein Mass	without RP-LC separation		after RP-LC separation		Annotation/ Homologue
		Hit count[a]	Relative abundance (Hit count /Protein Mass)	Hit count[a]	Relative abundance (Hit count /Protein Mass)	
m.73426	25287.5	222	8.78E-03	424	1.68E-02	uL2
m.73466	25896.9	222	8.57E-03	425	1.64E-02	uL2
m.73430	28704.4	224	7.80E-03	532	1.85E-02	uL2
m.254749	21423.4	138	6.44E-03	183	8.54E-03	uS7
m.306008	15514.8	95	6.12E-03	306	1.97E-02	eL27
m.69646	13451.5	78	5.80E-03	N/A	N/A	uL3
m.305692	18824.4	98	5.21E-03	63	3.35E-03	eL21
m.345032	17745.3	91	5.13E-03	262	1.48E-02	uS11
m.343677	31755	162	5.10E-03	401	1.26E-02	eS6
m.70448	31897.6	162	5.08E-03	164	5.14E-03	eS4
m.524763	29522.7	149	5.05E-03	140	4.74E-03	eS1
m.507694	30613.2	150	4.90E-03	N/A	N/A	uL18
m.442590	24861.8	111	4.46E-03	210	8.45E-03	eS7
m.157557	29780.5	129	4.33E-03	3	1.01E-04	uL30
m.116404	23628.1	98	4.15E-03	256	1.08E-02	eSEug1
m.454815	25749.8	105	4.08E-03	58	2.25E-03	uS3
m.117639	16842.1	68	4.04E-03	96	5.70E-03	uS12
m.514340	19223.4	76	3.95E-03	2	1.04E-04	uS17
m.342837	21340.4	84	3.94E-03	201	9.42E-03	eL20
m.484703	41867.5	159	3.80E-03	523	1.25E-02	uL4
m.111884	12378.2	46	3.72E-03	246	1.99E-02	eL36
m.261792	17184.5	63	3.67E-03	211	1.23E-02	uS9
m.265084	14422.1	51	3.54E-03	265	1.84E-02	eL29
m.487796	21973.6	75	3.41E-03	70	3.19E-03	uL6
m.17810	11736.1	40	3.41E-03	54	4.60E-03	eL43
m.17816	11736.1	40	3.41E-03	54	4.60E-03	eL43
m.484668	30033.2	100	3.33E-03	276	9.19E-03	uL4
m.447397	15078.5	49	3.25E-03	139	9.22E-03	eS12
m.154353	26961	86	3.19E-03	N/A	N/A	uS2
m.265086	16021.5	51	3.18E-03	264	1.65E-02	eL29
m.116398	32653.3	103	3.15E-03	360	1.10E-02	eSEug1
m.239117	18936.8	59	3.12E-03	480	2.53E-02	eL24
m.154112	23138.4	71	3.07E-03	16	6.91E-04	uS4
m.456163	23039.7	70	3.04E-03	3	1.30E-04	uL5
m.301457	26735.9	78	2.92E-03	1	3.74E-05	eL14
m.397219	26480.7	74	2.79E-03	437	1.65E-02	eL13
m.456342	15794.3	44	2.79E-03	130	8.23E-03	uL29
m.326399	7631.95	21	2.75E-03	13	1.70E-03	eS19
m.99611	17612.3	48	2.73E-03	83	4.71E-03	eS17
m.69549	46214.9	120	2.60E-03	N/A	N/A	uL3
m.321200	14855.6	38	2.56E-03	97	6.53E-03	eL34
m.51260	18534.5	47	2.54E-03	500	2.70E-02	uL23
m.106955	11979.2	30	2.50E-03	191	1.59E-02	P2
m.113554	30369.4	74	2.44E-03	250	8.23E-03	uS5

m.113564	30372.5	74	2.44E-03	250	8.23E-03	uS5
m.69324	18513.6	45	2.43E-03	158	8.53E-03	eS10
m.243191	15626.4	36	2.30E-03	154	9.86E-03	uL14
m.487189	25595.5	53	2.07E-03	59	2.31E-03	eL19
m.301460	19995.4	41	2.05E-03	1	5.00E-05	eL14
m.106973	37385.7	74	1.98E-03	27	7.22E-04	uL10
m.107013	32903.2	65	1.98E-03	17	5.17E-04	uL10
m.425579	17251.1	34	1.97E-03	219	1.27E-02	eS19
m.496416	6096.16	12	1.97E-03	70	1.15E-02	Similar to HABP4_PAI-RBP1 multi-domain protein (RNA-binding protein)
m.292284	23655.1	46	1.94E-03	11	4.65E-04	eL18
m.487258	29024.5	56	1.93E-03	59	2.03E-03	eL19
m.106937	11457.6	22	1.92E-03	17	1.48E-03	P1
m.425559	14687.8	27	1.84E-03	177	1.21E-02	eS19
m.110534	11451.9	21	1.83E-03	181	1.58E-02	eS21
m.514862	8819.83	16	1.81E-03	11	1.25E-03	N/A
m.154322	28421.8	50	1.76E-03	N/A	N/A	uS2
m.524701	14323	25	1.75E-03	164	1.15E-02	uS10
m.37956	6325.34	11	1.74E-03	N/A	N/A	N/A
m.242532	9447.44	16	1.69E-03	188	1.99E-02	eL38
m.189693	36028.9	61	1.69E-03	2	5.55E-05	eS8
m.189725	36028.9	61	1.69E-03	2	5.55E-05	eS8
m.459395	26317.1	44	1.67E-03	N/A	N/A	eL15
m.112065	17489.8	29	1.66E-03	318	1.82E-02	uL24
m.450931	16714.1	27	1.62E-03	140	8.38E-03	uL1
m.189713	23538.8	38	1.61E-03	N/A	N/A	eS8
m.347067	25687.6	41	1.60E-03	12	4.67E-04	uL16
m.129455	18952.3	30	1.58E-03	162	8.55E-03	uS13
m.451024	24680.4	39	1.58E-03	200	8.10E-03	uL1
m.462895	17181.5	27	1.57E-03	17	9.89E-04	uS19
m.462907	17181.5	27	1.57E-03	17	9.89E-04	uS19
m.323162	32803.7	51	1.55E-03	18	5.49E-04	N/A
m.141102	23277	36	1.55E-03	15	6.44E-04	eL6
m.242930	18779.9	29	1.54E-03	208	1.11E-02	uL22
m.242959	18779.9	29	1.54E-03	208	1.11E-02	uL22
m.460679	32898.6	50	1.52E-03	265	8.06E-03	eL8
m.217985	9541.9	14	1.47E-03	117	1.23E-02	eS27
m.242552	11078.4	16	1.44E-03	188	1.70E-02	eL38
m.103646	23060	33	1.43E-03	N/A	N/A	N/A
m.141131	25246	36	1.43E-03	14	5.55E-04	eL6
m.213214	16359	23	1.41E-03	22	1.34E-03	eL32
m.189732	16611.2	23	1.38E-03	2	1.20E-04	eS8
m.189766	16611.2	23	1.38E-03	2	1.20E-04	eS8
m.21245	16728.2	23	1.37E-03	196	1.17E-02	uS15
m.514333	5836.88	8	1.37E-03	N/A	N/A	uS17
m.50583	13161.4	18	1.37E-03	113	8.59E-03	eS25
m.300126	19657.5	26	1.32E-03	248	1.26E-02	uL11
m.241984	21298.4	27	1.27E-03	118	5.54E-03	eL22
m.182542	13474.5	17	1.26E-03	31	2.30E-03	N/A

m.172030	17015.4	21	1.23E-03	2	1.15E-04	uL4
m.21250	18824.3	23	1.22E-03	196	1.04E-02	uS15
m.368073	9999.46	12	1.20E-03	103	1.03E-02	eS28
m.450934	10090.3	12	1.19E-03	27	2.68E-03	uL1
m.507647	18643.1	21	1.13E-03	N/A	N/A	uL18
m.482897	19784.4	21	1.06E-03	267	1.35E-02	eS31
m.490855	14248.6	15	1.05E-03	49	3.44E-03	eL37
m.50113	35617.5	35	9.83E-04	N/A	N/A	RACK1
m.103626	58241.5	56	9.62E-04	N/A	N/A	N/A
m.57129	12534.7	12	9.57E-04	153	1.22E-02	ubiquitin
m.103637	60571.6	56	9.25E-04	N/A	N/A	N/A
m.147255	7582.8	7	9.23E-04	31	4.09E-03	uS14
m.149466	11095.8	10	9.01E-04	N/A	N/A	N/A
m.72489	13046.8	11	8.43E-04	239	1.83E-02	eL30
m.267991	21412.7	18	8.41E-04	117	5.46E-03	N/A
m.172036	25800.1	21	8.14E-04	2	7.63E-05	uL4
m.451021	15120.2	12	7.94E-04	60	3.97E-03	uL1
m.191411	18112.7	14	7.73E-04	1	5.52E-05	uL15 var1
m.50126	31331.4	23	7.34E-04	N/A	N/A	RACK1
m.137641	18165.5	13	7.16E-04	195	1.07E-02	eL31
m.243016	21002.7	15	7.14E-04	131	6.24E-03	uL22
m.243029	21002.7	15	7.14E-04	131	6.24E-03	uL22
m.292750	14032.4	10	7.13E-04	47	3.35E-03	eS26
m.50159	16866.3	12	7.11E-04	N/A	N/A	RACK1
m.343593	21605.6	15	6.94E-04	246	1.14E-02	eS6
m.343467	46358.1	32	6.90E-04	N/A	N/A	N/A
m.343605	24653.4	17	6.90E-04	263	1.07E-02	eS6
m.343652	24653.4	17	6.90E-04	263	1.07E-02	eS6
m.496439	34105.8	23	6.74E-04	371	1.09E-02	Similar to HABP4_PAI-RBP1 multi-domain protein (RNA-binding protein)
m.302171	39049.8	26	6.66E-04	66	1.69E-03	elongation factor 2
m.42141	7748.17	5	6.45E-04	N/A	N/A	N/A
m.42145	7748.17	5	6.45E-04	N/A	N/A	N/A
m.67596	94492.8	59	6.24E-04	N/A	N/A	N/A
m.432203	6881.66	4	5.81E-04	N/A	N/A	N/A
m.111192	13987.9	8	5.72E-04	213	1.52E-02	eL33
m.302015	17511.6	10	5.71E-04	86	4.91E-03	eS24
m.303893	38305.6	21	5.48E-04	13	3.37E-04	eS19
m.191417	18468.9	10	5.41E-04	2	1.08E-04	uL15 var1
m.303739	39541.2	21	5.31E-04	9	2.28E-04	eS19
m.303774	39541.2	21	5.31E-04	9	2.28E-04	eS19
m.303995	39541.2	21	5.31E-04	9	2.28E-04	eS19
m.191408	18840.1	10	5.31E-04	2	1.06E-04	uL15 var1
m.116425	22715.5	12	5.28E-04	254	1.12E-02	eSEug1
m.191356	18474.8	9	4.87E-04	N/A	N/A	uL15 var2
m.328430	20710	10	4.83E-04	N/A	N/A	uL13
m.328416	25244	12	4.75E-04	N/A	N/A	uL13
m.161032	44716.7	20	4.47E-04	N/A	N/A	uL3
m.51001	40480.1	18	4.45E-04	16	3.95E-04	N/A

m.294690	16070.5	7	4.36E-04	22	1.37E-03	uS8
m.301467	53614.8	23	4.29E-04	64	1.19E-03	SMC_N superfamily protein
m.303841	49213.4	21	4.27E-04	13	2.63E-04	eS19
m.303685	50449	21	4.16E-04	9	1.78E-04	eS19
m.289398	19249.9	8	4.16E-04	47	2.44E-03	Similar to Arginine methyltransferase-interacting protein (AIR1 superfamily protein)
m.308977	19295.7	8	4.15E-04	35	1.81E-03	m.308977 cognate
m.308983	19295.7	8	4.15E-04	35	1.81E-03	m.308977 cognate
m.102563	39950.7	16	4.00E-04	1	2.50E-05	dihydrolipoyl transacetylase
m.161064	50937.8	20	3.93E-04	N/A	N/A	uL3
m.161090	50937.8	20	3.93E-04	N/A	N/A	uL3
m.212840	17864.7	7	3.92E-04	88	4.93E-03	eukaryotic translation initiation factor 5A
m.127626	12856.5	5	3.89E-04	N/A	N/A	uL4
m.102553	41807.7	16	3.83E-04	1	2.39E-05	dihydrolipoyl transacetylase
m.89092	19062.7	7	3.67E-04	N/A	N/A	N/A
m.275142	37170.2	13	3.50E-04	N/A	N/A	uS2
m.102577	46039	16	3.48E-04	1	2.17E-05	dihydrolipoyl transacetylase
m.50990	52074.2	18	3.46E-04	16	3.07E-04	N/A
m.242991	6021.11	2	3.32E-04	6	9.96E-04	uL22
m.243046	6021.11	2	3.32E-04	6	9.96E-04	uL22
m.103620	25818	8	3.10E-04	N/A	N/A	N/A
m.252685	6530.83	2	3.06E-04	N/A	N/A	N/A
m.284311	13100.8	4	3.05E-04	2	1.53E-04	N/A
m.74573	13793.2	4	2.90E-04	71	5.15E-03	Similar to Alba domain-containing protein (DNA/RNA-binding protein albA)
m.74585	13793.2	4	2.90E-04	71	5.15E-03	Similar to Alba domain-containing protein (DNA/RNA-binding protein albA)
m.74709	13793.2	4	2.90E-04	71	5.15E-03	Similar to Alba domain-containing protein (DNA/RNA-binding protein albA)
m.191397	7376.06	2	2.71E-04	2	2.71E-04	N/A
m.130894	18640.5	5	2.68E-04	N/A	N/A	N/A
m.87742	7501.12	2	2.67E-04	N/A	N/A	N/A
m.363075	23624.2	6	2.54E-04	N/A	N/A	N/A
m.56984	7993.03	2	2.50E-04	8	1.00E-03	N/A
m.365895	12148.4	3	2.47E-04	N/A	N/A	N/A
m.322106	12204.3	3	2.46E-04	1	8.19E-05	N/A
m.191402	8228.63	2	2.43E-04	2	2.43E-04	N/A
m.308162	8449.83	2	2.37E-04	287	3.40E-02	eS30
m.180311	33983.6	8	2.35E-04	N/A	N/A	N/A
m.454111	28299.5	6	2.12E-04	N/A	N/A	N/A
m.317610	14361.8	3	2.09E-04	N/A	N/A	N/A
m.363079	29166.9	6	2.06E-04	N/A	N/A	N/A
m.454154	95023.1	19	2.00E-04	N/A	N/A	N/A
m.64670	16165.4	3	1.86E-04	43	2.66E-03	Similar to NAC domain-containing protein

m.12384	10896	2	1.84E-04	8	7.34E-04	N/A
m.476663	5591.93	1	1.79E-04	N/A	N/A	N/A
m.362475	28185.8	5	1.77E-04	26	9.22E-04	N/A
m.343455	28267.8	5	1.77E-04	N/A	N/A	N/A
m.227938	5767.13	1	1.73E-04	N/A	N/A	RACK1
m.34980	5913.3	1	1.69E-04	N/A	N/A	N/A
m.142471	17833.8	3	1.68E-04	N/A	N/A	N/A
m.484893	12362.3	2	1.62E-04	N/A	N/A	N/A
m.398035	6703.51	1	1.49E-04	N/A	N/A	N/A
m.329304	34529.7	5	1.45E-04	N/A	N/A	N/A
m.497686	42369.7	6	1.42E-04	N/A	N/A	EGR-2
m.497699	42369.7	6	1.42E-04	N/A	N/A	EGR-2
m.329812	35329.4	5	1.42E-04	13	3.68E-04	N/A
m.162344	7267.72	1	1.38E-04	N/A	N/A	N/A
m.127292	7349.86	1	1.36E-04	N/A	N/A	N/A
m.329299	36996.8	5	1.35E-04	N/A	N/A	N/A
m.135938	14831.5	2	1.35E-04	N/A	N/A	N/A
m.90625	15174.6	2	1.32E-04	N/A	N/A	N/A
m.86691	45863.2	6	1.31E-04	N/A	N/A	N/A
m.223121	7794.23	1	1.28E-04	N/A	N/A	N/A
m.308902	31537.3	4	1.27E-04	N/A	N/A	N/A
m.250455	23888.8	3	1.26E-04	40	1.67E-03	N/A
m.143199	32817.1	4	1.22E-04	N/A	N/A	N/A
m.143244	32817.1	4	1.22E-04	N/A	N/A	N/A
m.498395	33230.3	4	1.20E-04	4	1.20E-04	N/A
m.19650	8558.26	1	1.17E-04	N/A	N/A	N/A
m.282103	26741.8	3	1.12E-04	N/A	N/A	N/A
m.386636	9020.63	1	1.11E-04	N/A	N/A	N/A
m.526652	9268.96	1	1.08E-04	49	5.29E-03	eLEgr2
m.111196	74241.9	8	1.08E-04	6	8.08E-05	N/A
m.111210	74241.9	8	1.08E-04	6	8.08E-05	N/A
m.111223	74241.9	8	1.08E-04	6	8.08E-05	N/A
m.66128	9336.03	1	1.07E-04	N/A	N/A	N/A
m.66283	9336.03	1	1.07E-04	N/A	N/A	N/A
m.452539	56132.8	6	1.07E-04	N/A	N/A	N/A
m.283598	9368.91	1	1.07E-04	N/A	N/A	N/A
m.257943	9870.93	1	1.01E-04	8	8.10E-04	m.257943 cognate
m.72226	7478.96	N/A	N/A	92	1.23E-02	eLEgr3
m.496451	10625.6	1	9.41E-05	102	9.60E-03	Similar to HABP4_PAI-RBP1 multi-domain protein (RNA-binding protein)
m.519165	8454.31	N/A	N/A	80	9.46E-03	eLEgr1
m.284337	12628.9	N/A	N/A	113	8.95E-03	N/A
m.50614	6614.72	N/A	N/A	31	4.69E-03	N/A
m.110258	6890.76	N/A	N/A	23	3.34E-03	N/A
m.110251	7370.96	N/A	N/A	23	3.12E-03	N/A
m.479328	27875.7	2	7.17E-05	78	2.80E-03	Similar to Alba domain-containing protein (DNA/RNA-binding protein albA)

m.479358	27875.7	2	7.17E-05	78	2.80E-03	Similar to Alba domain-containing protein (DNA/RNA-binding protein albA)
m.479329	29371.4	2	6.81E-05	78	2.66E-03	Similar to Alba domain-containing protein (DNA/RNA-binding protein albA)
m.185381	21769.9	N/A	N/A	57	2.62E-03	N/A
m.185387	21769.9	N/A	N/A	57	2.62E-03	m.185381 cognate
m.110254	9412.12	N/A	N/A	23	2.44E-03	N/A
m.163372	25807.2	1	3.87E-05	62	2.40E-03	N/A
m.41590	5433.6	N/A	N/A	13	2.39E-03	N/A
m.185370	24865.4	N/A	N/A	57	2.29E-03	m.185381 cognate
m.185418	24879.5	N/A	N/A	57	2.29E-03	m.185381 cognate
m.185301	25056.5	N/A	N/A	57	2.27E-03	m.185381 cognate
m.185413	25056.5	N/A	N/A	57	2.27E-03	m.185381 cognate
m.110262	10378.6	N/A	N/A	23	2.22E-03	N/A
m.110271	10617.6	N/A	N/A	23	2.17E-03	N/A
m.257303	32782.8	N/A	N/A	57	1.74E-03	N/A
m.257352	34267.4	N/A	N/A	57	1.66E-03	m.257303 cognate
m.257374	34423.5	N/A	N/A	57	1.66E-03	m.257303 cognate
m.106928	8616.49	N/A	N/A	14	1.62E-03	N/A
m.520491	11503	N/A	N/A	15	1.30E-03	Similar to NAC and UBA_NACA_NACP1 domain-containing protein
m.15966	10323.3	N/A	N/A	12	1.16E-03	N/A
m.108842	9741.33	N/A	N/A	11	1.13E-03	N/A
m.179493	5645.99	N/A	N/A	6	1.06E-03	N/A
m.167738	5855.06	N/A	N/A	6	1.02E-03	N/A
m.292136	28506.2	N/A	N/A	29	1.02E-03	N/A

[a.] The MS/MS spectra of peptides derived from the identified protein were selected with probability-based Mowse scores (total score) that exceeded its threshold indicating a significant homology ($p < 0.05$), and referred to them as ‘hits’. N/A, not assigned.

Supplementary Table S5| RNA modifications in *Euglena* ribosome

rRNA	Residue number	Modification type	Cryo-EM*	rRNA	Residue number	Modification type	Cryo-EM*
18S	8	Um	√	18S	1966	Um	√
	28	Am	√		2041	Um	√
	38	Cm	√		2046	Am	√
	40	Am	√		2075	Gm	√
	42	Gm	√		2123	Cm	√
	57	Gm	√		2142	Cm	√
	99	Cm	√		2180	Am	√
	103	Cm	√				
	110	Am	√		27	Ψ	
	179	Um	√		32	Ψ	
	180	Gm	√		89	Ψ	
	186	Um	√		105	Ψ	
	390	Gm	√		121	Ψ	
	393	Cm	√		176	Ψ	
	407	Am	√		280	Ψ	
	485	Gm	√		403	Ψ	
	533	Am	√		465	Ψ	
	565	Am	√		544	Ψ	
	621	Cm	√		640	Ψ	
	641	Gm	√		1068	Ψ	
	645	Am	√		1378	Ψ	
	649	Am	√		1393	Ψ	
	682	Um	√		1396	Ψ	
	702	Um	√		1554	Ψ	
	704	Um	√		1591	Ψ	
	723	Am	√		1592	Ψ	
	1037	Cm	√		1624	Ψ	
	1063	Am	√		1660	Ψ	
	1096	Am	√		1711	Ψ	
	1371	Am	√		1715	Ψ	
	1536	Gm	√		1960	Ψ	
	1539	Um	√		2065	Ψ	
	1549	Am	√		2081	Ψ	
	1616	Um	√		2101	Ψ	
	1625	Cm	√		2116	Ψ	
	1641	Um	√		2129	Ψ	
	1679	Um	√		2131	Ψ	
	1681	Gm	√		2305	Ψ	
	1705	Gm	√				
	1736	Gm	x		1597	$m^1\Psi$	√
	1744	Um	√		1601	$m^1acp^3\Psi$	√
	1861	Cm	x		2078	m^7G	√
	1900	Am	√		2144	m^5C	√
	1901	Gm	√		2287	m^6_2A	√
	1913	Gm	x		2288	m^6_2A	√

rRNA	Residue number	Modification type	Cryo-EM*
LSU 1 (5.8S)	39	Am	√
	41	Gm	√
	74	Gm	√
	97	Am	√
	16	Ψ	
	68	Ψ	
LSU 2	183	Am	√
	206	Um	√
	215	Gm	√
	247	Um	√
	234	Ψ	
	280	Ψ	
	281	Ψ	
LSU 3	483	Cm	√
	491	Um	√
	537	Cm	√
	541	Gm	√
	577	Cm	√
	594	Am	√
	628	Gm	√
	631	Gm	√
	302	Ψ	
	308	Ψ	
	421	Ψ	
	480	Ψ	
	498	Ψ	
	567	Ψ	
LSU 5	935	Am	√
	936	Cm	√
	949	Cm	√
	1039	Am	√
	1081	Um	√
	1104	Gm	√
	1106	Am	√
	1121	Am	√
	1165	Cm	√
	1185	Um	√
	1189	Um	√
	1201	Gm	√
	1204	Am	√
	1208	Gm	√
	1217	Gm	√

rRNA	Residue number	Modification type	Cryo-EM*
LSU 5	1218	Cm	√
	1222	Um	√
	1270	Um	√
	1312	Cm	√
	1315	Um	√
	1324	Gm	√
	1452	Um	√
	1023	Ψ	
	1075	Ψ	
	1118	Ψ	
	1126	Ψ	
	1171	Ψ	
	1184	Ψ	
	1198	Ψ	
	1235	Ψ	
	1260	Ψ	
	1266	Ψ	
	1363	Ψ	
	1365	Ψ	
	1407	Ψ	
	931	m^1A	√
	938	m^7G	√
LSU 6	1502	Am	√
	1504	Um	√
	1553	Um	√
	1562	Cm	x
	1573	Cm	√
	1608	Gm	x
	1624	Am	x
	1626	Cm	x
	1647	Cm	x
	1649	Cm	x
	1655	Cm	x
	1662	Gm	√
	1667	Gm	√
	1681	Um	√
	1683	Gm	√
	1738	Am	√
	1815	Am	√
	1819	Gm	√
	1822	Cm	√
	1824	Um	√
	1826	Gm	√
	1828	Um	√
	1834	Am	x

rRNA	Residue number	Modification type	Cryo-EM*
LSU 6	1835	Gm	√
	1836	Cm	√
	1856	Gm	√
	1883	Gm	√
	1888	Am	√
	1891	Am	√
	1898	Um	√
	1929	Am	√
	1956	Gm	√
	1957	Um	√
	1976	Cm	√
	1568	Ψ	
	1582	Ψ	
	1586	Ψ	
	1637	Ψ	
	1692	Ψ	
	1859	Ψ	
	1876	Ψ	
	1926	Ψ	
	1863	m^1A	x
	1864	m^1A	x
	1866	m^1A	x
	1868	m^1A	x
	1869	m^1A	x
	1923	m^3Cm	■
LSU 7	1999	Um	√
	2005	Am	√
	2009	Gm	√
	2022	Am	√
	2129	Um	√
	2146	Gm	√
	2119	Ψ	
	2125	Ψ	
	2154	Ψ	
	2171	Ψ	
LSU 8	2242	Cm	√
	2313	Cm	√
	2346	Um	√
	2348	Cm	√
	2349	Am	√
	2358	Am	√
	2602	Cm	√
	2605	Um	√

rRNA	Modified nucleotide	Type	Cryo-EM*	rRNA	Modified nucleotide	Type	Cryo-EM*	rRNA	Modified nucleotide	Type	Cryo-EM*
	2610	Gm	√		2915	Ψ			3145	Ψ	
	2625	Um	√		2943	Ψ			3167	Ψ	
	2685	Cm	√						3175	Ψ	
	2708	Am	√	LSU 8	2636	m^3C	■		3185	Ψ	
	2713	Um	√		2766	m^5C	√		3204	Ψ	
	2744	Am	√		2785	h_2U	x		3206	Ψ	
	2768	Am	√		2904	$m^1Ψ$	√		3235	Ψ	
	2769	Am	√		2971	Gm	x		3332	Ψ	
	2776	Gm	√		2979	Gm	x		3412	Ψ	
	2806	Um	√		2981	Am	x		3440	Ψ	
	2809	Am	√		2982	Gm	x		3444	Ψ	
	2812	Am	√		2988	Um	x	LSU 9	3446	Ψ	
	2814	Am	√		2993	Cm	x		3451	Ψ	
	2825	Cm	√		2996	Cm	x		3503	Ψ	
	2832	Cm	√		3085	Cm	x		3510	Ψ	
	2835	Um	√		3086	Cm	x		3531	Ψ	
	2849	Am	√		3159	Um	√		3542	Ψ	
	2853	Cm	√		3171	Um	√		3562	Ψ	
	2870	Gm	√		3187	Gm	√		3568	Ψ	
	2879	Gm	√		3191	Gm	√				
	2883	Gm	√		3208	Cm	√		3214	m^6A	√
	2887	Am	√		3222	Cm	√		3456	m^5C	√
	2897	Gm	√		3223	Gm	√		3518	m^5C	√
	2909	Um	√		3300	Um	√		3540	m^3U	√
LSU 8	2920	Am	√		3315	Am	√				
	2925	Gm	√		3347	Cm	√		3636	Um	√
	2953	Gm	x		3348	Am	√		3688	Gm	√
				LSU 9	3351	Um	x		3694	Um	√
	2331	Ψ			3355	Cm	√		3718	Um	√
	2361	Ψ			3367	Am	√				
	2586	Ψ			3374	Cm	√	LSU 10	3591	Ψ	
	2591	Ψ			3377	Gm	√		3644	Ψ	
	2617	Ψ			3379	Gm	√		3680	Ψ	
	2621	Ψ			3401	Gm	√		3697	Ψ	
	2623	Ψ			3434	Gm	√		3701	Ψ	
	2624	Ψ			3465	Cm	√				
	2642	Ψ			3500	Am	√		3906	Am	√
	2679	Ψ			3504	Gm	√				
	2742	Ψ			3508	Um	√	LSU 12	3865	Ψ	
	2746	Ψ			3509	Gm	√				
	2752	Ψ			3521	Am	√		3957	Cm	
	2754	Ψ			3533	Am	√				
	2802	Ψ			3535	Cm	√	LSU 13	3953	Ψ	
	2837	Ψ			3546	Cm	√		3963	Ψ	
	2842	Ψ									
	2874	Ψ			2970	Ψ		LSU 14	3969	Ψ	
	2899	Ψ			2999	Ψ					
	2914	Ψ			3042	Ψ					

* With two exceptions, the listed modifications are those previously identified and reported based on direct chemical and enzymatic mapping (1). MS was able to identify two LSU rRNA residues that were not definitively identified in (1): *C1923 as m^3Cm and *C2636 as m^3C (Cm?2794 in (1) was found to be an unmodified C). Modifications independently confirmed by cryo-EM and /or MS are indicated by ■.

* Modifications confirmed by cryo EM are indicated by √.

Supplementary Table S6| Conservation of *E. gracilis* rRNA modifications

E. gracilis	*E. coli*	*T. thermophilus*	*S. cerevisiae*	*L. donovani*	*H. sapiens*
**SSU**					
Um8				Um8	
Ψ27					
Am28			Am28	Am28	Am27
Y32					
Cm38				Cm38	
Am40					
Gm42					
Gm57					
Y89					
Cm99			Am100	Am98	Am99
Cm103				Ψ104	
Ψ105			Ψ106		Ψ105
Am110					
Ψ121					Ψ121
Ψ176					
Um179					
Gm180					Am166
Um186					Um172
Ψ280					
Gm390					
Cm393					
Ψ403					
Am407					
Ψ465					
Gm485					Gm436
Am533			Am436	Am479	Am484
Ψ544					
Am565					Am512
Cm621					
Ψ640					
Gm641					
Am645					Am590
Am649					
Um682			Um578		Um627
Um702					
Um704					ψ649
Am723			Am619	Am668	Am668
Cm1037					
Am1063					
Ψ1068					
Am1096					
Am1371			Am974		Am1031
Ψ1378					
Ψ1393					
Ψ1396			Ψ999	Ψ1396	Ψ1056
Gm1536			Gm1126	Gm1478	
Um1539					
Am1549					
Ψ1554					
Ψ1591				Ψ1533	Ψ1238
Ψ1592					
m^1Ψ1597			Ψ1187		Um1244
m^1acp^3Ψ1601	m^2G966	m^2G944	m1acp3 Ψ1191	m^1acpψ1543	m^1acpψ1543
Um1616					
Ψ1624				Ψ1566	
Cm1625					Cm1272
Um1641					Um1288
Ψ1660					
Um1679[a]			Um1269	Um1621	Um1326
Gm1681			Gm1271	Gm1623	Gm1328

Gm1705				Gm1647	
Ψ1711					
Ψ1715				Ψ1657	
Gm1736				Am1383	
Um1744					Cm1931
Cm1861					
Am1900			Ψ1415		
Gm1901					
Gm1913			Gm1428	Gm1829	Gm1490
Ψ1960					
Um1966					
Um2041					
Am2046					
Ψ2065					
Gm2075			Gm1572		
m^7G2078[b]			m^7G1575	m^7G1995	m^7G1639
Ψ2081					
Ψ2101					
Ψ2116					
Cm2123					
Ψ2129				Ψ2046	
Ψ2131				Um2048	Ψ1692
Cm2142	m^4Cm1402	m^4Cm1402	Cm1639	m^4Cm2059	Cm1703
m^5C2144				m^5C2061	
Am2180					
m^6_2A2287[c]	m^6_2A1517	m^6_2A1496	m^6_2A1781	m^6_2A2184	m^6_2A1850
m^6_2A2288[c]	m^6_2A1518	m^6_2A1497	m^6_2A1782	m^6_2A2185	m^6_2A1851
Ψ2305					
**LSU**					
Ψ16[d]					
Am39					
Gm41				Am43(5.8)	
Ψ68				Ψ69(5.8)	Ψ69(5.8)
Gm74				Gm75(5.8)	Gm75(5.8)
Am97					
Am183					
Um206					
Gm215					
Ψ234					
Um247					
Ψ280					
Ψ281					
Ψ302					
Ψ308					
Ψ421					
Ψ480					
Cm483					
Um491					
Ψ498					
Cm537					
Gm541					
Ψ567					
Cm577					
Am594					Am400
Gm628					
Gm631					
m^1A931			m^1A645		m^1A1322
Am935			Am649	Am681(α)	Am1326
Cm936			Cm650		
m^7G938					
Cm949			Cm663	Cm695(α)	Cm1340
Ψ1023					
Am1039					
Ψ1075			Ψ776		

Um1081			
Gm1104	Gm805	Gm856(α)	Gm1522
Am1106	Am807	Am858(α)	Am1524
Ψ1118		Ψ870(α)	Ψ 1536
Am1121			
Ψ1126			
Cm1165			
Ψ1171			
Ψ1184			
Um1185			
Um1189		Ψ940(α)	
Ψ1198	Um898		
Gm1201			
Am1204		Am955(α)	
Gm1208[e]	Gm908	Gm959(α)	Gm1625
Gm1217			
Cm1218[f]			
Um1222			
Ψ1235			
Ψ1260	Ψ960	Ψ1011(α)	Ψ1677
Ψ1266	Ψ966	Ψ1017(α)	Ψ1683
Um1270			
Cm1312			
Um1315			
Gm1324			
Ψ1363			Ψ1779
Ψ1365			Ψ1781
Ψ1407			
Um1452			
Am1502	Am1133	Gm1190(α)	Am1871
Um1504			
Um1553			
Cm1562			
Ψ1568			
Cm1573			
Ψ1582			
Ψ1586			
Gm1608			
Am1624			
Cm1626			
Ψ1637			
Cm1647			
Cm1649			
Cm1655			
Gm1662			
Gm1667			
Um1681		Um1371(α)	
Gm1683			
Ψ1692			
Am1738			
Am1815			
Gm1819		Gm1526(α)	
Cm1822	Cm1437	Cm1529(α)	Cm2351
Um1824			
Gm1826			
Um1828		Ψ1535(α)	
Am1834	Am1449	Am1541(α)	Am2363
Gm1835	Gm1450		Gm2364
Cm1836			Cm2365
Gm1856			
Ψ1859			
m^1A1863			
m^1A1864			
m^1A1866			

m^{1}A1868					
m^{1}A1869					
Ψ1876					
Gm1883					
Am1888					
Am1891					
Um1898					
m^{3}Cm1923					Cm2422
Ψ1926					
Am1929					
Gm1956					
Um1957					
Cm1976					
Um1999					
Am2005					
Gm2009					
Am2022					
Ψ2119					
Ψ2125					Ψ2632
Um2129					
Gm2146					
Ψ2154					
Ψ2171					
Cm2242					
Cm2313					Cm2804
Ψ2331					
Um2346			Um1888	Gm71(β)	Um2837
Cm2348				Cm73(β)	Um2839
Am2349					
Am2358					
Ψ2361					
Ψ2586					
Ψ2591					
Cm2602					
Um2605					
Gm2610					
Ψ2617			Ψ2129		
Ψ2621			Ψ2133		Ψ3637
Ψ2623					Ψ3639
Ψ2624				Am382(β)	
Um2625					
m^{3}C2636					
Ψ2642					
Ψ2679			Ψ2191	ψ437(β)	Ψ3695
Cm2685			Cm2197	Cm443(β)	Cm3701
Am2708			Am2220		Am3724
Um2713					
Ψ2742	ψ1911	ψ1932		Ψ500(β)	Ψ3758
Am2744			Am2256	Am502(β)	Am3760
Ψ2746	m^3 ψ1915	m^{5}U1936	Ψ2258	Ψ504(β)	Ψ3762
Ψ2752			Ψ2264	Ψ510(β)	Ψ3764
Ψ2754			Ψ2266	Ψ512(β)	
m^{5}C2766			m^{5}C2278	Gm534(β)	m^{5}C3782
Am2768			Am2280		
Am2769			Am2281		Am3785
Gm2776			Gm2288		Gm3792
h$_2$U2785					
Ψ2802			Ψ2314	Um560(β)	Ψm3818
Um2806					Ψ3822
Am2809					Am3825
Am2812				Am570(β)	
Am2814				Am572(β)	Am3830
Cm2825			Cm2337	Cm583(β)	Cm3841
Cm2832					

Um2835			Ψm2347	Ψ593(β)	Ψ3851
Ψ2837			Ψ2349	Ψ595(β)	Ψ3853
Ψ2842					
Am2849					
Cm2853				Ψ611(β)	Cm3869
Gm2870				Am628(β)	
Ψ2874					
Gm2879					
Gm2883				Gm641(β)	Gm3899
Am2887					
Gm2897				Gm655(β)	
Ψ2899	m^{7}G2069				
m^{1}Ψ2904			Ψ2416	Ψ662(β)	Ψ3920
Um2909			Um2421	Um667(β)	Um3925
Ψ2914					
Ψ2915					
Am2920					
Gm2925					
Ψ2943					
Gm2953					
Ψ2970					
Gm2971					
Gm2979					
Am2981					
Gm2982					
Um2988					
Cm2993					
Cm2996					
Ψ2999					
Ψ3042					
Cm3085					
Cm3086					
Ψ3145					
Um3159		Gm2262			
Ψ3167					
Um3171					
Ψ3175					
Ψ3185					
Gm3187					
Gm3191	Gm2251		Gm2691	Gm1047(β)	Gm4196
Ψ3204					
Ψ3206			m^{3}U2634		
Cm3208					
m^{6}A3214					
Cm3222				Um1078(β)	Um4227
Gm3223				Gm1079(β)	Gm4228
Ψ3235					
Um3300					
Am3315					
Ψ3332					
Cm3347					
Am3348					
Um3351					
Cm3355					
Am3367					
Cm3374					
Gm3377			Gm2791	Gm1230(β)	
Gm3379			Gm2793	Gm1232(β)	Gm4370
Gm3401			Gm2815	Gm1254(β)	Gm4392
Ψ3412	ψ2457		Ψ2826	Ψ1265(β)	Ψ4403
Gm3434					
Ψ3440					Ψ4431
Ψ3444					
Ψ3446					

Ψ3451			Ψ2865	Ψ1304(β)	Ψ4442
m^5C3456			m^5C2870		m^5C4447
Cm3465				Cm1318(β)	Cm4456
Am3500					
Ψ3503				Ψ1355(β)	Ψ4493
Gm3504					Gm4494
Um3508	Um2552	Um2563	Um2921	Um1360(β)	Um4498
Gm3509			Gm2922	Gm1361(β)	Gm4499
Ψ3510			Ψ2923	Ψ1362(β)	Ψ4500
m^5C3518					
Am3521				Am1373(β)	
Ψ3531			Ψ2944	Ψ1383(β)	Ψ4521
Am3533			Am2946	Am1385(β)	Am4523
Cm3535			Cm2948		
m^3U3540					m^3U4530
Ψ3542[j]					Ψ4532
Cm3546			Cm2959	Cm1938(β)	Cm4536
Ψ3562			Ψ2975	Ψ1414(β)	Ψ4552
Ψ3568				Um1420(β)	
Ψ3591					Ψ4579
Um3636				Gm74(δ)	
Ψ3644					
Ψ3680					
Gm3688					
Um3694					
Ψ3697					
Ψ3701					Ψ4689
Um3718					
Ψ3865					
Am3906					
Ψ3953					Ψ5010
Cm3957					
Ψ3963					
Ψ3969					

Supplementary Table S7|RNA modifications detected by MS.

rRNA	Residue number	Modification type determined by MS/MS/MS	RNase T1/A fragment: sequence determined by MS/MS	RNase T1/A fragment: (Candidate) position(s)	Note
LSU 6	1863	m^1A	$C(m^1A)(m^1A)C(m^1A)C(m^1A)(m^1A)CCCCGp$	LSU6: 1862_1874	MS/MS sequence analysis showed that all 5 adenosines in the fragment have a methylated base. The MS/MS/MS spectrum of the bases produced m^1A-specific signals at both m/z 106.029 and 107.036. No signals at m/z 92.026 and/or 106.041 specific for m^2A, m^6A and m^8A were detected.
LSU 6	1864	m^1A			
LSU 6	1866	m^1A			
LSU 6	1868	m^1A			
LSU 6	1869	m^1A			
LSU 6	1923	m^3Cm	$(m^3Cm)CGp$	LSU6: 1655_1657, 1923_1925[a]	MS/MS sequence analysis showed that C1923 has two methyl residues. The MS/MS spectrum contains specific signals for $O^{2'}$-methylation at m/z 225.04 and monomethylated cytosine at m/z 124.052, demonstrating the residue is $O^{2'}$-methylated and base-monomethylated cytidine. The MS/MS/MS spectrum of the base produced m^3C-specific signals at m/z 67.030.
LSU 8	2636	m^3C	$(m^3C)AGCp$	LSU8: 2414_2417, 2636_2639[a], 2653_2656, 2715_2718, 2877_2880	MS/MS sequence analysis showed that C2636 has a methyl residue. The MS/MS spectrum contains a specific signal for monomethylated cytosine at m/z 124.052. The MS/MS/MS spectrum of the base produced m^3C-specific signals at m/z 67.031.

[a.] Most plausible position deduced from cryo-electron microscopic data and previous chemical mapping (1).

References

1. Schnare, M.N. and Gray, M.W. (2011) Complete modification maps for the cytosolic small and large subunit rRNAs of *Euglena gracilis*: Functional and evolutionary implications of contrasting patterns between the two rRNA components. *J. Mol. Biol.*, **413**, 66-83.

2.. Afonine, P., Headd, J., Terwilliger, T. and Adams, P. (2013) New tool: phenix. real_space_refine. *Computational Crystallography Newsletter*, **4**, 43-44.

3. Chen, V.B., Arendall, W.B., Headd, J.J., Keedy, D.A., Immormino, R.M., Kapral, G.J., Murray, L.W., Richardson, J.S. and Richardson, D.C. (2010) MolProbity: all-atom structure validation for macromolecular crystallography. *Acta Crystallographica Section D: Biological Crystallography*, **66**, 12-21.
